# A Detailed Analysis of the *BR*_1_ Locus Suggests a New Mechanism for Bolting after Winter in Sugar Beet (*Beta vulgaris* L.)

**DOI:** 10.3389/fpls.2016.01662

**Published:** 2016-11-14

**Authors:** Conny Tränkner, Ioana M. Lemnian, Nazgol Emrani, Nina Pfeiffer, Surya P. Tiwari, Friedrich J. Kopisch-Obuch, Sebastian H. Vogt, Andreas E. Müller, Markus Schilhabel, Christian Jung, Ivo Grosse

**Affiliations:** ^1^Plant Breeding Institute, University of KielKiel, Germany; ^2^Institute of Computer Science, Martin Luther University Halle-WittenbergHalle, Germany; ^3^Institute of Clinical Molecular Biology, University of KielKiel, Germany; ^4^German Centre for Integrative Biodiversity Research (iDiv) Halle-Leipzig-JenaLeipzig, Germany

**Keywords:** sugar beet, winter beet, flowering time, mapping-by-sequencing, vernalization

## Abstract

Sugar beet (*Beta vulgaris* ssp. vulgaris) is a biennial, sucrose-storing plant, which is mainly cultivated as a spring crop and harvested in the vegetative stage before winter. For increasing beet yield, over-winter cultivation would be advantageous. However, bolting is induced after winter and drastically reduces yield. Thus, post-winter bolting control is essential for winter beet cultivation. To identify genetic factors controlling bolting after winter, a F_2_ population was previously developed by crossing the sugar beet accessions BETA 1773 with reduced bolting tendency and 93161P with complete bolting after winter. For a mapping-by-sequencing analysis, pools of 26 bolting-resistant and 297 bolting F_2_ plants were used. Thereby, a single continuous homozygous region of 103 kb was co-localized to the previously published *BR*_1_ QTL for post-winter bolting resistance (Pfeiffer et al., 2014). The *BR*_1_ locus was narrowed down to 11 candidate genes from which a homolog of the *Arabidopsis CLEAVAGE AND POLYADENYLATION SPECIFICITY FACTOR 73-I* (*CPSF73-I*) was identified as the most promising candidate. A 2 bp deletion within the BETA 1773 allele of *BvCPSF73-Ia* results in a truncated protein. However, the null allele of *BvCPSF73-Ia* might partially be compensated by a second *BvCPSF73-Ib* gene. This gene is located 954 bp upstream of *BvCPSF73-Ia* and could be responsible for the incomplete penetrance of the post-winter bolting resistance allele of BETA 1773. This result is an important milestone for breeding winter beets with complete bolting resistance after winter.

## Introduction

Sugar beet (*Beta vulgaris* ssp. *vulgaris* var. *altissima*) is the only sucrose-storing crop cultivated in temperate regions. It accounts for nearly 30% of the world's annual sugar production (http://faostat3.fao.org, 2015), whereby pulp and molasses are used for animal feeding and methane production. Sugar beets are conventionally sown in spring and harvested in the vegetative stage before winter. Cultivation of sugar beet as a winter crop instead, by sowing in autumn and harvesting in the next year, might increase the beet yield up to 26% due to the pre-winter development and the accelerated growth in spring (Jaggard and Werker, 1999; Hoffmann and Kluge-Severin, 2011). The advanced development of winter beets will also allow an earlier harvest and start of beet campaigns. Therefore, winter beet production is one of the major aims in sugar beet breeding.

One challenge of winter cultivation is the control of bolting after winter. Sugar beets are biennials that grow vegetatively during the first season. A prolonged exposure to cold during winter results in vernalization and the plants acquire floral competence. Under long-day conditions of the next season, the plants start bolting which is indicated by stem elongation and is followed by flower development. Bolting drastically reduces the beet and sugar yield and hampers harvesting processes (Wood and Scott, 1975; Jaggard et al., 1983; Hoffmann and Kluge-Severin, 2011). Thus, bolting is completely undesired for farming although necessary for seed production. To transform sugar beet from a summer into a winter crop, bolting control is an obligate requirement.

Sugar beets were bred for obligate vernalization requirement, whereas wild beets include annual, biennial, and even perennial plants (Hautekeete et al., 2001; Van Dijk, 2009). In *Arabidopsis thaliana*, the gene *FLOWERING LOCUS C* (*FLC*) is a major regulator of vernalization requirement. Although the *FLC*-homolog of beet, *BvFL1*, could complement *Arabidopsis flc* mutants, it does not play a major role in controlling the vernalization response of biennial beets (Reeves et al., 2007; Vogt et al., 2014). Instead, the gene *BOLTING TIME CONTROL 1* (*BTC1*) that resides within the *B* locus of *B. vulgaris* is a major regulator of vernalization requirement (Pin et al., 2012). Beets with a dominant *BTC1* allele behave as annuals and require only long-day conditions for floral transition. Beets homozygous for the recessive *btc1* alleles behave as biennials and require cold exposure followed by long-days to bolt. A second gene, *BvBBX19*, was recently identified from the *B2* locus, which controls the vernalization requirement of beets epistatically to *BTC1* (Dally et al., 2014). *BTC1* and *BvBBX19* promote annual growth through repression of the *B. vulgaris* bolting repressor gene *BvFT1* and activation of the floral activator gene *BvFT2*, both homologs of the floral integrator gene *FLOWERING LOCUS T* (*FT*) of *Arabidopsis*. Plants with homozygous recessive alleles of either *BTC1* or *BvBBX19* express *BvFT1* and suppress *BvFT2* resulting in bolting resistance before winter. Subsequent vernalization leads to *BvFT1* down-regulation and *BvFT2* activation and bolting is promoted (Pin et al., 2012; Dally et al., 2014). Vernalization and subsequent bolting is also epigenetically controlled in beets. Recent studies showed that cold exposure alters the DNA and RNA methylation in shoot apical meristems of sugar beets resulting in gene expression patterns specific for bolting sensitive and resistant genotypes. Thereby, the DNA methylation correlates negatively with the bolting rate and DNA hypermethylation treatments delay or even inhibit bolting in vernalized sugar beets (Trap-Gentil et al., 2011; Hébrard et al., 2013, 2016).

An obligate bolting tolerance or resistance after winter is required for winter beet cultivation. Biennial beets, that over-express *BvFT1* or repress *btc1* expression, show bolting resistance after vernalization (Pin et al., 2010, 2012). Within the *Beta* gene pool, natural variation for post-winter bolting resistance is also available. Kirchhoff et al. (2012) identified beet accessions with low bolting tendencies (e.g., BETA 1773) after growing wild and cultivated beet accessions over winter. Recently, a major quantitative trait locus (QTL) for post-winter bolting resistance (*BR*_1_) was determined using a F_3_ mapping population derived from a cross between BETA 1773 and a sugar beet which is regularly bolting after winter (Pfeiffer et al., 2014). Both parents were homozygous for the biennial *btc1* allele and the annual *BBX19* allele. *BTC1* and *BvBBX19* control early flowering resulting in an annual or biennial life cycle. In contrast, *BR*_1_ controls bolting after winter. Plants homozygous for the recessive allele (*br*_1_) cannot bolt even after winter which in the following will be termed “never-bolting” or “post-winter bolting resistance.” Within the F_2_ and F_3_ generations, post-winter bolting resistance showed a non-Mendelian segregation. Thus, post-winter bolting resistance was determined as a quantitative trait which was measured as bolting rates of F_3_ families. The QTL was mapped to chromosome 9 with a confidence interval of 4 cM and explained 65% of the phenotypic variation. Thereby, the BETA 1773 allele caused a reduced bolting rate in a partially recessive manner (Pfeiffer et al., 2014). F_3_ families that were homozygous for the BETA 1773 allele at the *BR*_1_ locus showed an average bolting rate of 0.33, indicating an incomplete penetrance of the bolting resistance allele. Although the *BR*_1_ locus was genetically mapped and quantitatively characterized, the genetic factor which underlies *BR*_1_ and its interaction with other bolting genes are unknown.

As shown recently for different organisms, genetic regions controlling discrete phenotypes can be mapped by applying Next Generation Sequencing (NGS) on pooled DNA of individuals that were bulked by their discrete phenotypes. By aligning NGS reads of the distinct sequence bulks to a reference genome and comparing read allele frequencies between both sequence bulks, trait-related loci can be identified and screened for candidate genes (Schneeberger et al., 2009; Laitinen et al., 2010; Qi et al., 2013; Takagi et al., 2013; Lu et al., 2014; Mascher et al., 2014). The publication of the sugar beet genome now allows the application of mapping-by-sequencing also for *B. vulgaris* (Dohm et al., 2014).

In this study, we aimed to physically map the *BR*_1_ locus and to identify *BR*_1_ candidate genes by applying mapping-by-sequencing. To achieve this, we used the same F_2_ population that was developed to genetically map the *BR*_1_ QTL (Pfeiffer et al., 2014). This population segregated for bolting behavior after cold-treatment. DNA of bolting-resistant and bolting F_2_ plants were bulked to produce a bolting-resistant and a bolting sequence pool. We hypothesized that bolting-resistant plants were homozygous for *br*_1_, whereas bolting plants were either heterozygous or homozygous for the bolting or even bolting resistance allele due to the incomplete penetrance of *br*_1_ (*BR*_1_*BR*_1_, *br*_1_*br*_1_). Accordingly, the bolting sequence pool was expected to be polymorphic at each position that has a cross-specific sequence variation, even at the *BR*_1_ locus. In contrast, the bolting-resistant sequence pool was expected to be monomorphic at the *br*_1_ locus whereas any other genomic region, which does not control the bolting-resistant phenotype, was expected to be polymorphic. Applying a sliding window analysis, we physically mapped the *BR*_1_ locus to a 103 kb region on chromosome 9. This region encompasses 11 genes that we analyzed for sequence polymorphisms and characterized for putative functions. A putative function of the most promising *BR*_1_ candidate gene, a *CPSF73-I* homolog of *Arabidopsis*, is discussed.

## Materials and methods

### Plant material and growth conditions

According to Pfeiffer et al. (2014), a F_2_ population consisting of 410 plants was derived from a single F_1_ plant that was produced by a hand-cross of the biennial sugar beets BETA 1773 and 93161P (Figure S1). Seeds of BETA 1773 and 93161P were provided by the Leibniz Institute of Plant Genetics and Crop Plant Research, IPK (Gatersleben, Germany) and the breeding company Saatzucht Dieckmann (Nienstädt, Germany), respectively. BETA 1773 segregates for bolting and never-bolting plants, whereas 93161P plants are regularly bolting after winter. Subsequently we named BETA 1773 “P_br_” and the bolting parent 93161P “P_b._” Both accessions are homozygous for the biennial *btc1* allele and the dominant *BvBBX19* allele (*btc1*_*a*_/*btc1*_*a*_, *BvBBX19*/*BvBBX19*). As described in detail by Pfeiffer et al. (2014), the F_2_ plants were pre-cultivated in the greenhouse from November 1 to December 20, 2010 under 16 h light at 20°C. Then they were cold-treated for 16 weeks in a cold chamber at 5°C and under 22 h light. After 1 week acclimatization under 22 h light and 8°C, the F_2_ plants were planted on April 19, 2011 to a field nursery in Kiel, Germany. Bolting F_2_ plants were bag-isolated to produce F_3_ seeds. On December 9, 2011, seeds of 254 F_3_ families were sown in soil in quickPot-plates96T (Hermann Meyer KG, Germany) and cultivated at 20°C and 16 h light in the greenhouse for 4 weeks. Subsequent cold treatment was done for 16 weeks at 5°C and 16 h light in a climate chamber. At the beginning of May 2012, 248 F_3_ families were planted to field nurseries in Kiel, Germany (Pfeiffer et al., 2014). The bolting phenotype was recorded on October 11, 2011 for F_2_ plants and on October 15, 2012 for F_3_ plants. Bolting was scored, when stem elongation was visible. Furthermore, the existence of flowers and floral buds was recorded as an indicator for complete floral transition.

For climate chamber experiments, 93161P and two F_3_ families were used. The F_3_ families were homozygous for the recessive *br*_1_ allele as determined previously for the corresponding parental F_2_ plant using marker CAU3903. The plants were cultivated in soil in 9 × 9 × 9.5 cm^3^ pots in the greenhouse at 16 h light and 22°C. After 4.5 weeks, 45–47 plants per accession were transferred to cold chambers and kept under 16 h light at 4 and 6°C, respectively. After 14 weeks of cold-treatment, 22–24 plants from the 4 and 6°C cold-treatment were kept in climate chambers under 22 or 16 h light at 20°C for 3 months.

### DNA extraction and sequencing

Genomic DNA was extracted from freeze-dried leaf samples of the parental, F_1_ and F_2_ plants following a slightly modified CTAB protocol (Saghai-Maroof et al., 1984). DNA amount and quality were determined on 1% agarose gels against λ DNA standards (http://www.thermofisher.com) and using the NanoDrop 2000 spectrophotometer (http://www.thermofisher.com). DNA concentration was adjusted to 10 ng/μl.

### NGS and analysis of NGS data

DNA was pooled based on the plant's phenotype (bolting or bolting-resistant after cold-treatment). Four NGS libraries (B0679–B0682) with DNA from 6 to 7 bolting-resistant plants and two NGS libraries (B0683, B0684) with DNA from 148 to 149 bolting plants, respectively, were prepared for whole genome sequencing to generate a bolting-resistant (“br pool”) and bolting sequence pool (“b pool”). Each library was sequenced with the Illumina HiSeq2000 system (http://www.illumina.com) at one lane as 101 bp paired-end reads (Table S1). The raw sequence data have been deposited at the NCBI in the Short Read Archive (SRA) database under the accession number SRP078892. Quality control was performed with FastQC (http://www.bioinformatics.babraham.ac.uk/projects/fastqc/). The raw data were trimmed using Sickle (https://github.com/najoshi/sickle) using the paired end mode with 25 as threshold for trimming based on average quality in a window and 60 nucleotides as minimal length for accepted reads after removing low quality bases. High quality paired-end reads were mapped to the sugar beet reference genome RefBeet-1.1 (Dohm et al., 2014) using Bowtie 2 version 2.1.0 (Langmead and Salzberg, 2012) with the parameters—sensitive-local—X 800–I 100. To reduce ambiguity in the alignment, reads with multiple mappings were filtered out. An InDel realignment and duplicate removal was performed on uniquely mapped reads using GATK (McKenna et al., 2010). Afterwards, a multi-sample SNP/InDel and genotype calling was done with GATK's UnifiedGenotyper version 2.8 (DePristo et al., 2011; Van der Auwera et al., 2013). Only SNPs and InDels (≤24 nucleotides) at positions with at least 50-fold coverage over all DNA pools were kept for further analysis. A SNP or InDel position was considered as candidate if it was heterozygous within the b pool and homozygous within the br pool. The first condition was necessary to exclude sequence polymorphisms due to differences to the reference genome derived from the sugar beet genotype KWS2320. For the sliding window analysis, we used a window size of 200 kb and a step size of 100 kb. Per window, the number of monomorphic positions of the br pool out of polymorphic positions of the b pool was computed using in-house R scripts.

A second computational processing of the same NGS raw data was performed using the software CLC Genomics Workbench 6.5.1 (http://www.clcbio.com) to validate specific sequence polymorphisms of *BR*_1_ candidate genes based on two independently generated read mapping sets. The 101 bp paired-end reads were imported with a paired-end distance of 50–1000 nucleotides. After quality control, 2 nucleotides were removed from the 5′ site of all reads. Reads were only accepted with maximum 2 ambiguous nucleotides and a minimum length of 80 nucleotides. Trimmed paired and broken reads were mapped against the reference genome RefBeet-1.1 with mismatch cost 2, insertion and deletion cost 1, length fraction 0.5 and similarity fraction 0.9. Only uniquely mapped reads were considered.

Translation of nucleotide sequences was done using the CLC software Main Workbench 7.5 or Genomics Workbench 6.5.1 (http://www.clcbio.com). For candidate genes, a gene ontology analysis was performed using Blast2GO (https://www.blast2go.com). The miRNA target prediction was done using the web server TAPIR (Bonnet et al., 2010). BLAST analyses of DNA and protein sequences were performed using the bl2seq function of NCBI BLAST (http://blast.ncbi.nlm.nih.gov/Blast.cgi). Multiple sequence alignments were performed using Clustal Omega 1.2.1 (Sievers et al., 2011).

### Molecular marker analysis

Molecular markers were developed based on sequence polymorphisms detected in NGS read mapping data. Primers flanking these polymorphic regions were designed using the software tool OligoCalc (Kibbe, 2007). All primers were obtained from Eurofins Genomics (http://www.eurofinsgenomics.eu). Polymerase chain reaction (PCR) assays were run in a total volume of 15 μl consisting of 1 × PCR buffer without Mg^2+^, 1.5 mM MgCl_2_, 0.2 mM dNTP mix, 0.2 mM of forward and reverse primer, 0.3 U Taq DNA Polymerase (Invitrogen™, http://www.thermofisher.com) and up to 10 ng template DNA. PCR products were separated by agarose gel electrophoresis or Sanger sequenced (Institute for Clinical Molecular Biology/IKMB, University of Kiel, Germany). All markers including the primer sequences are listed in Table S2.

## Results

### A physical map of the post-winter bolting resistance locus

**Figure 1:**
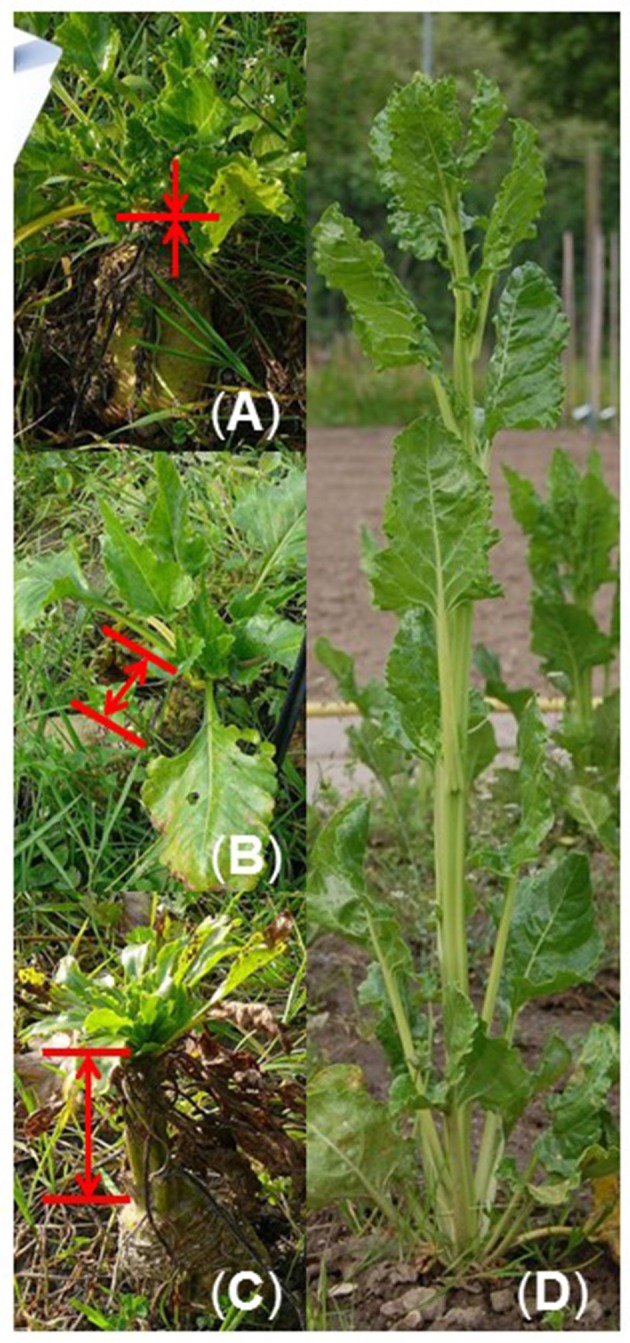
**Bolting and bolting-resistant F_**2**_ beets in the field after cold-treatment. (A)** Bolting-resistant beet growing vegetatively without stem elongation. **(B,C)** Beets with incomplete bolting producing stem-like structures of 5–50 cm (marked by arrows) but lacking inflorescences and flowers. **(D)** Normally bolting beet from the bolting accession 93161P with elongated stem, regular inflorescence, and flower development.

For mapping post-winter bolting resistance, a sugar beet F_2_ population was used that segregated for bolting and bolting resistance after cold-treatment. This population consisted of 410 F_2_ individuals and was derived from a cross between BETA 1773 (P_br_) and 93161P (P_br_, Figure S1). After cold-treatment, the F_2_ population segregated into 26 bolting-resistant, 365 regularly bolting, and 19 plants with incomplete bolting. Plants, that showed incomplete bolting, produced stem-like structures from 5 to 50 cm without developing flowers (Figure 1). The 26 bolting-resistant plants were classified as post-winter bolting resistant, whereas the 19 plants with incomplete bolting were classified as bolting together with the 365 regular bolting beets.

To physically map post-winter bolting resistance, we selected the 26 bolting-resistant F_2_ plants and produced 4 NGS libraries (B0679–B0682, br pool) each containing DNA of 6–7 bolting-resistant plants (Table S3). In addition, we produced two more NGS libraries (B0683, B0684, b pool) which contained DNA of 148 and 149 randomly selected bolting F_2_ plants. Each library was subjected to high-throughput sequencing on an Illumina HiSeq system, which resulted in 6 sequence sub-pools that contained in total 1140 million 2 × 101 bp paired-end reads corresponding to 230 Gbp of sequence data. Trimmed reads of each sub-pool were mapped separately to the sugar beet reference genome RefBeet-1.1 (Dohm et al., 2014). For each library, between 31 and 33% of the trimmed reads mapped uniquely (including paired and single reads), resulting in 14–25-fold coverage of the published genome sequence (Table S1). In sum, a 72-fold and 34-fold coverage of the RefBeet-1.1 sequence were obtained for the br and b pool, respectively. Through multi-sample SNP/InDel and genotype calling of the uniquely mapped reads of each sub-pool against RefBeet-1.1, we detected in total 3,576,287 positions with sequence polymorphisms in at least one of the 6 sequence sub-pools.

Next, the data of the two b sub-pools were combined and used to identify cross-specific sequence polymorphisms. On Refbeet-1.1 positions with cross-specific sequence polymorphisms, the read allele frequencies were about 0.5, because the b pool contained a genome-wide mixture of P_br_ and P_b_ sequences at each genome position due to bulking F_2_ plants. Positions with cross-specific polymorphisms were computed as heterozygous by genotype calling. In contrast, monomorphic positions of the b pool indicated a sequence variation to RefBeet-1.1 with identical sequences of the crossing parents. In total, 2,105,427 positions with heterozygous, cross-specific polymorphisms were identified.

**Figure 2:**
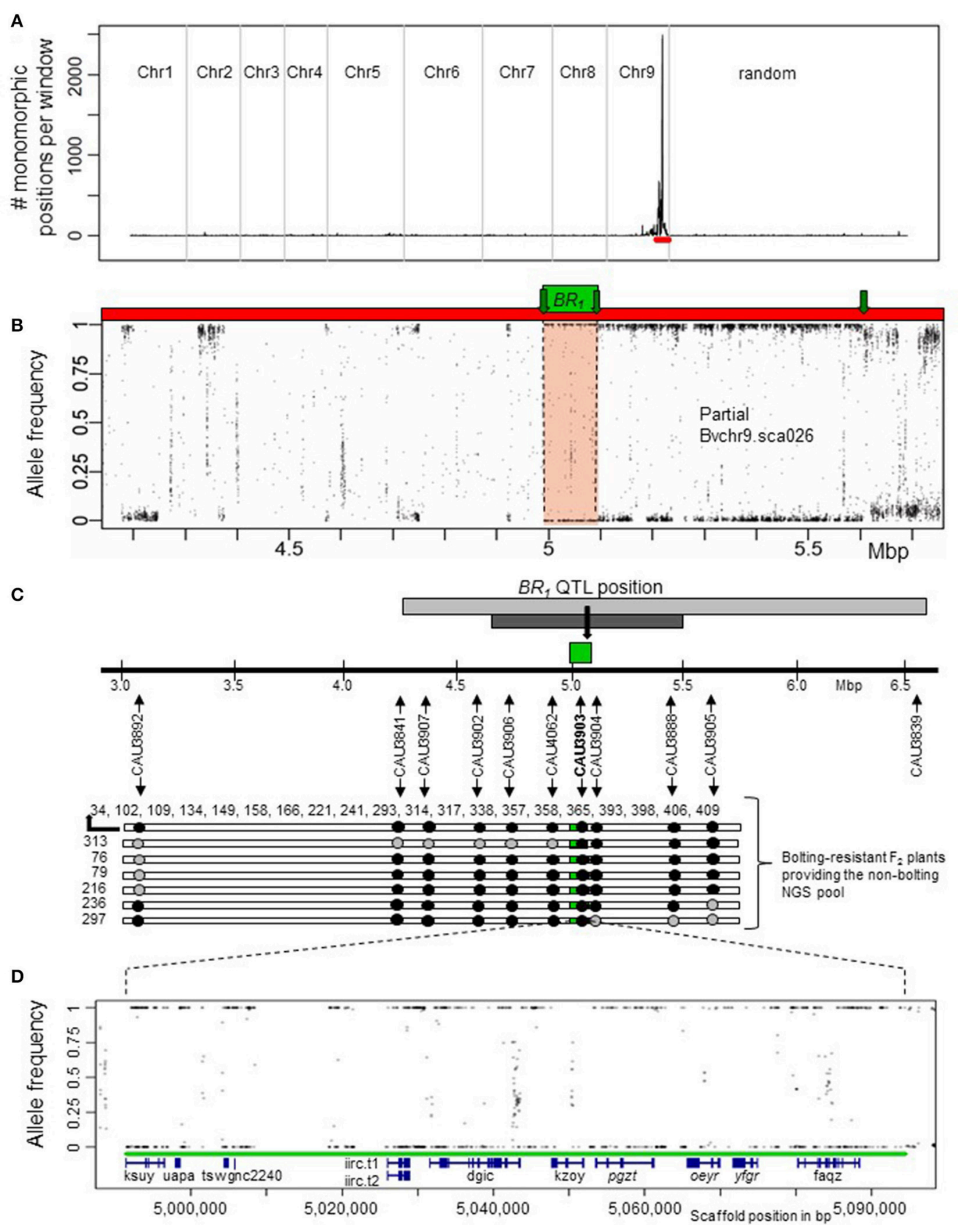
**Localization of the ***BR***_**1**_ locus by a mapping-by-sequencing strategy. (A)** A major peak was detected on chromosome 9 at scaffold Bvchr9.sca026 (red bar) by plotting the number of monomorphic positions of the br pool out of polymorphic positions of the b pool within a 200 kb sliding window and a step size of 100 kb. **(B)** Allele frequencies in the br pools based on polymorphic positions in the b pool at scaffold Bvchr9.sca026 (red). The genome-wide screening had revealed only a single, 103 kb region (green box) that was completely monomorphic in the br pool (allele frequency values 1 and 0 indicate monomorphic positions different and identical to RefBeet-1.1, respectively) at positions that were polymorphic in the b pool. This region is located at Bvchr9.sca026 between position 4,991,549 and 5,094,401. For the remaining genome positions, the allele frequencies differed from 1 and 0, whereby positions at unlinked regions showed on average allele frequencies of about 0.5 (not shown). Green arrows indicate recombination sites of bolting-resistant F_2_ plants. **(C)** Co-localization of the *BR*_1_ QTL and the physically mapped *BR*_1_ locus (green). Localization of the *BR*_1_ QTL (light gray), its confidence interval (dark gray) and QTL position (black arrow) is based on the sequence of the QTL flanking markers CAU3841 and CAU3839 (Pfeiffer et al., 2014) in relation to RefBeet-1.1. Genotypic data derived from 10 codominant CAU markers at the *BR*_1_ locus indicate crossover events around the *BR*_1_ locus in 6 out of 26 bolting-resistant F_2_ plants. The plant IDs of 20 plants homozygous for *BR*_1_: 34, 102, 109, 134, 149, 158, 166, 221, 241, 293, 314, 317, 338, 357, 358, 365, 393, 398, 406, 409. Black arrows show marker positions, black dots indicate marker positions that are homozygous for the allele derived from the bolting-resistant parent BETA 1773, gray dots indicate heterozygous positions. **(D)** Location of RefBeet-1.1 gene models (blue) within the physically mapped *BR*_1_ locus (green): ksuy.t1, uapa.t1, tswg.t1, nc2240, iirc.t1/t2, dgic.t1, kzoy.t1, pgzt.t1, oeyr.t1, yfgr.t1, faqz.t1. Details are given in Table 1. Genes in reverse orientation are written in italics.

Subsequently, we searched within the br pool for continuous homozygous regions. For this, we applied a sliding window approach over all scaffolds and contigs of RefBeet-1.1. In this way, we analyzed all positions that were called heterozygous in the b pool and called homozygous in the br pool. As a result, only a single peak on chromosome 9 at scaffold Bvchr9.sca026 was detected (Figure 2A), indicating that only one major locus controls post-winter bolting resistance. Bvchr9.sca026 is located at the bottom of chromosome 9, where the *BR*_1_ QTL had been previously mapped (Pfeiffer et al., 2014). To locate the candidate region on Bvchr9.sca026, we plotted the average read allele frequency of the br pool at each position that was called heterozygous in the b pool (Figure 2B). Next, the graphic was screened for continuous regions showing allele frequencies of 1 and 0, which indicate homozygous-different or homozygous-identical positions compared to RefBeet-1.1. A continuous homozygous region with a length of 103 kb was detected between scaffold positions 4,991,549 and 5,094,401 (Figure 2B). The *BR*_1_ QTL flanking markers CAU3841 and CAU3839 are located 0.75 Mbp upstream and 1.47 Mbp downstream of the identified 103 kb-region (Figure 2C). Thus, the QTL interval covers the complete region. Combining physical and genetic mapping data, we reason that we have physically mapped the *BR*_1_ locus.

Following, we confirmed the physically mapped *BR*_1_ region and the flanking recombination sites by marker analysis. We selected the *BR*_1_-specific marker CAU3903 and 9 flanking markers (Figure 2C), and genotyped the 26 bolting-resistant F_2_ plants. All 26 plants were homozygous for the bolting resistance allele *br*_1_ derived from P_br_ (Figure 2D, Table S3). By using the flanking markers, we detected several crossover events within this population. The bolting-resistant F_2_ plant 313 carried the first crossover at the left side of the *BR*_1_ locus, followed by crossovers of plant 76, 79, and 216. At the right side, the first and second crossovers were detected for plant 297 and 236, respectively (Figure 2D). These results confirmed the map position of the *BR*_1_ locus and proved that post-winter bolting resistance was derived from the bolting-resistant accession BETA 1773.

### Low penetrance of the *BR*_1_ allele for post-winter bolting resistance

The physical mapping of the *BR*_1_ locus was based on 26 bolting-resistant F_2_ plants. To determine the distribution of the bolting resistance allele *br*_1_ within the whole F_2_ population, we genotyped all 410 F_2_ plants using the co-dominant marker CAU3903 which is specific for the *BR*_1_ locus. We detected 95 plants that were homozygous for *br*_1_, 201 heterozygous plants and 114 plants homozygous for the dominant bolting allele *BR*_1_ (Table S4). The marker genotypes segregated 1:2:1 (*X*^2^ = 1.917, non-significant at α = 0.05). This segregation ratio is typical for F_2_ populations and shows that no artificial selection had occurred favoring one of the alleles. Out of 95 F_2_ plants that were genotyped as homozygous for *br*_1_, only 26 plants were bolting-resistant after cold treatment, whereas 13 and 56 plants showed incomplete or regular bolting, respectively. In contrast, all plants with a dominant *BR*_1_ allele were bolting. Thus, the genotype at the *BR*_1_ locus predicted 83% of the phenotype. However, 73% of the F_2_ plants that were homozygous for *br*_1_ were bolting which could be explained by further loci which might be involved in bolting control after winter. Thus, we performed additional genome wide screenings under various parameter settings using the sequence information of the 26 bolting-resistant F_2_ plants. However, we did not detect other loci that could explain the bolting resistance phenotype assuming that the *BR*_1_ locus might interact with various minor loci that are below the detection level.

**Figure 3:**
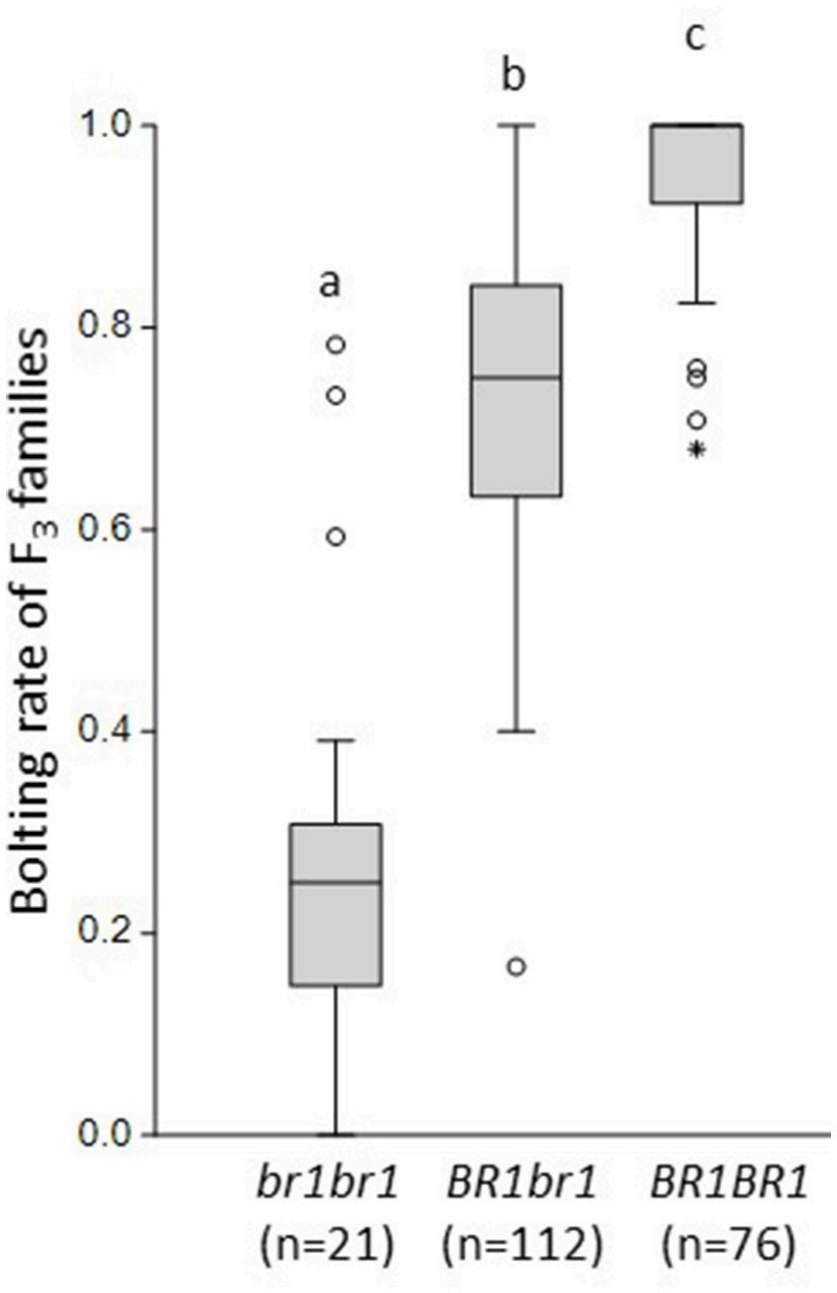
**Bolting rates of F_**3**_ families grouped by the ***BR***_**1**_ genotypes of their F_**2**_ parents**. Marker CAU3903 was used for genotyping. The mean bolting rates of F_3_ families differed significantly between all *BR*_1_ genotypes. Statistical analysis is based on Tukey's pairwise test with α = 0.05. The letters a, b, c indicate significant differences.

The F_2_ phenotypic data are based on single plants. Next, we evaluated the post-winter bolting resistance of 248 F_3_ families that were derived from selfed F_2_ plants as described by Pfeiffer et al. (2014). After cold treatment, the F_3_ plants grew under field conditions from May until October 2012, together with control plants of the bolting-resistant and bolting accessions. Post-winter bolting resistance was recorded and the bolting rate was calculated for each F_3_ family as the number of bolting plants out of the total plant number of this family. The bolting-resistant control showed a bolting rate of 0.12, whereas the bolting control bolted completely. Under the same growing conditions, 209 F_3_ families with 6–31 plants per family showed bolting rates between 0 and 1. Depending on the F_2_ genotype at the *BR*_1_ locus, the average bolting rates of these F_3_ families varied significantly (Figure 3). F_3_ families derived from F_2_ plants that were homozygous for the *br*_1_ allele showed an average bolting rate of 0.27 ± 0.216. F_3_ families derived from heterozygous or homozygous-dominant F_2_ plants showed average bolting rates of 0.74 ± 0.146 and 0.96 ± 0.075, respectively. These results demonstrate a clear dominant-recessive inheritance of post-winter bolting resistance which is controlled primarily by the *BR*_1_ locus. Only regularly bolting F_2_ plants could be used to produce F_3_ families. Noteworthy, 3 out of 21 regularly bolting F_2_ plants that were homozygous for *br*_1_, generated completely bolting-resistant F_3_ families (in sum 71 plants). This suggests a reduced penetrance of the *br*_1_ allele rather than additional *BR* loci.

Moreover, we used F_3_ phenotypic data to explore if the *BR*_1_ locus also controls incomplete bolting of sugar beets resulting in stem-like structures without producing flowers. 183 out of 209 F_3_ families contained 1–18 plants that had bolted incompletely until the end of the season. When grouping the F_3_ families according to the *BR*_1_ genotype, then 71.4 (homozygous *br*_1_), 97.3 (heterozygous) and 77.6% (homozygous *BR*_1_) of the F_3_ families included incomplete bolting plants. Thus, the incomplete bolting phenotype cannot be explained through the presence of a certain *BR*_1_ allele and genetic factors unlinked to *BR*_1_ seem to control this phenotype.

**Figure 4:**
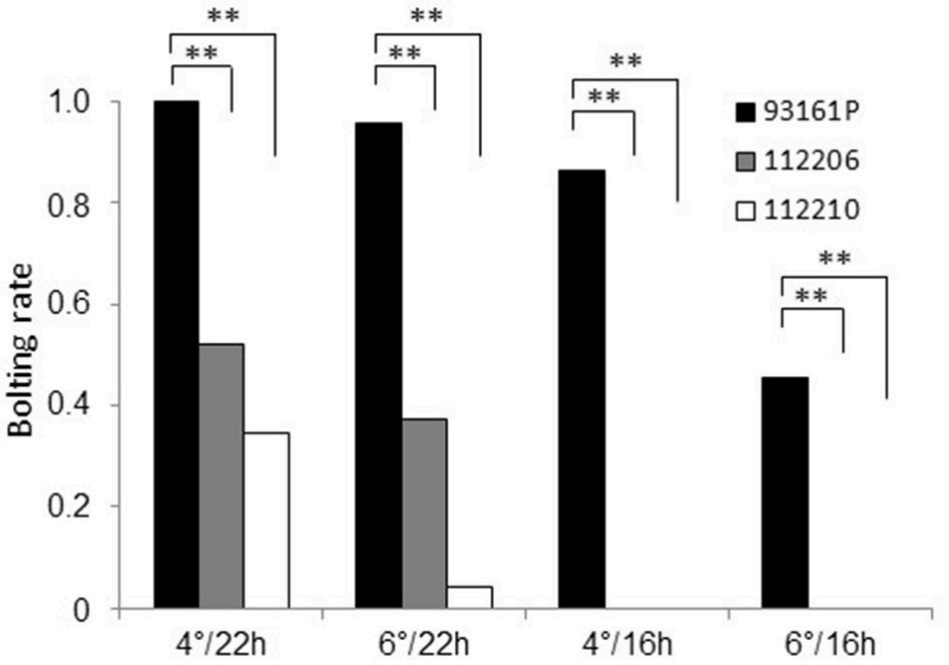
**Temperature and day-length dependence of bolting rates of F_**3**_ families 112206 and 112210 which are homozygous for ***br***_**1**_**. The parental accession 93161P was used as bolting control. Plants were vernalized at 4°C or 6°C under 16 h light and subsequently grown under 22 or 16 h light at 20°C. Asterisks indicate significant differences at p.adjust < 0.05 for each growth condition based on Pearson's chi-squared tests.

Subsequently we aimed to determine the effect of vernalization temperature and photoperiod on the bolting rate of plants which were homozygous for *br*_1_. We selected two F_3_ families (112206, 112210) that were derived from *br*_1_ homozygous F_2_ plants. These plants were cold-treated together with bolting control plants at 4° or 6°C, to test whether different vernalization temperatures affect the bolting rate. Subsequently, the plants were kept at 20°C under 22 h or 16 h light, to test whether different photoperiods affect the bolting rate. After 3 months, we determined the bolting rate per accession and treatment. The bolting rates of the F_3_ families ranged from 0.52 to 0 (Figure 4). They were significantly reduced compared to the bolting control in each treatment. Under 22 h light, the bolting rates ranged from 0.52 to 0.35 after vernalization at 4°C and from 0.38 to 0.04 after 6°C vernalization, respectively. Complete bolting resistance was obtained under 16 h light in both families for both cold treatments. The bolting control showed also a reduced bolting rate under sub-optimal conditions of decreased day-length and increased vernalization temperature. Thus, environmental factors such as day length and vernalization intensity affect bolting after vernalization, whereby the impact of the photoperiod was significantly stronger than the impact of vernalization temperature. Although both F_3_ families responded with varying bolting rates to different growth conditions, they never reached a bolting rate of 1 due to the *br*_1_-mediated bolting resistance.

### The *BR*_1_ locus houses 11 genes

Based on the RefBeet-1.1 annotation, we found 11 gene models with RNA evidence within the *BR*_1_ locus including 10 protein-coding and one miRNA gene (Table 1). However, the gene ontology (GO) analysis identified no genes or miRNA target genes related to floral transition genes or flowering time genes of *Arabidopsis*. We expected for the *BR*_1_ gene a P_br_ specific mutation that results in a reduced or complete loss-of-function. Furthermore, the *BR*_1_ gene should be expressed in leaves and/or shoot apices where plants perceive floral inducing signals (Corbesier and Coupland, 2006). In a first step, we screened the coding sequences of the 11 genes for nucleotide variations between the parents and RefBeet-1.1. For this, we used the NGS read mapping sets of the bolting-resistant and bolting sequence pools and a more stringent read mapping of the sequence sub-pools B0679 and B0680 (bolting-resistant) and B0683 (bolting). No sequence variation was found for the miRNA gene. For the protein-coding genes, we detected 7 genes with P_br_ specific amino acid changes, which resulted in 3 genes (uapa.t1, tswg.t1, yfgr.t1) with premature STOP codons (Table 1, Figure S2). Thereby, yfgr.t1 showed the most severe mutation through a 2 bp deletion that causes a frameshift. This frameshift results in a truncated protein with a length of 203 instead of 633 amino acids. The P_br_ specific sequence polymorphisms in uapa.t1 and tswg.t1 resulted in less truncated proteins with lengths of 226 instead of 237 and 190 instead of 204 amino acids.

**Table 1:** **Characterization of ***B. vulgaris*** genes located within the ***BR***_**1**_ locus interval (102,853 bp) on chromosome 9 on scaffold Bvchr9.sca026**.

**Gene models based on RefBeet-1.1 (abbreviation)**	**P_br_ (BETA 1773) specific amino acid changes compared to RefBeet-1.1 and P_b_ (93161P)**	**GO protein description/putative function**	**Homologous *Arabidopsis* gene, annotation according to TAIR database**
Bv9_227290_ksuy.t1 (ksuy)^a^^,^^b^^,^^c^	No	Curvature thylakoid protein, chloroplastic	AT1G52220, *CURVATURE THYLAKOID 1C* (*CURT1C*), protein located in the chloroplast thylakoid membrane
Bv9_227300_uapa.t1 (uapa)^a^	aa51 H 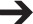 D, aa119 T 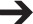 S, aa208 T 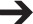 A, premature STOP codon for P_br_ and P_b_ resulting in a protein with 223 instead of 235aa (RefBeet-1.1)	DNA-directed RNA polymerase II subunit RPB1-like	No significant hit
Bv9_227310_tswg.t1 (tswg)^a^	aa84 T 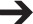 K, aa128 H 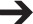 -, aa151 P 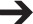 PS, aa182 P 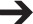 L, aa187 F 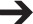 L, aa188 D 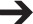 F, aa189 H 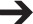 STOP codon resulting in a protein with 188 instead of 202aa	Hypothetical protein BVRB_9g221070	No significant hit
Bv9_227320_iirc.t1/t2 (iirc)^a^^,^^b^	aa204 E 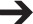 Q, aa255 A 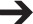 E, aa300 E 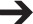 G, aa319 Q 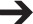 R, aa332 A 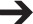 V, aa339 R 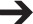 S, aa407/373 K 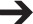 N, aa454/420 V 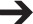 I, aa467/433 F 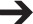 S specific for t1 and t2, aa384 I 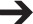 V specific for t1	Serpin-ZX-like protein (F: serine-type endopeptidase inhibitor activity, transferase activity)	AT1G47710, *SERPIN*, protein involved in the negative regulation of endopeptidase activity in the apoplast
Bv9_227330_dgic.t1 (dgic)^a^^,^^b^^,^^c^	aa95 E 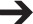 G, aa113 V 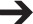 L, aa152 E 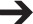 D, aa223 T 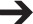 S, aa248 G 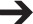 E, aa252 K 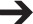 N, aa266 V 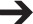 I, aa302 I 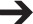 T, aa309 Q 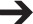 L, aa324 A 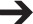 V, aa772 E 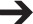 D, aa787 A 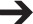 S	Golgin candidate 5	AT1G79830, *GOLGIN CANDIDATE 5* (*GC5*), structural component of the Golgi apparatus
Bv9_227340_kzoy.t1 (kzoy)^a^^,^^b^^,^^c^	No	Basic-leucine zipper transcription factor family protein isoform 1 (F:sequence-specific DNA binding transcription factor activity; P:regulation of transcription, DNA-dependent)	AT1G58110, basic-leucine zipper (bZIP) transcription factor, protein involved in DNA-templated transcription in the nucleus
Bv9_227350_pgzt.t1 (pgzt)^a^^,^^b^^,^^c^	No	Magnesium transporter mrs2-4 (C: membrane; F:magnesium ion transmembrane transporter activity; P:transmembrane transport, magnesium ion transport)	AT3G58970, *MAGNESIUM TRANSPORTER 6* (*MGT6*), integral component of the plasma membrane
Bv9_227360_oeyr.t1 (oeyr)^a^^,^^b^^,^^c^	aa618 E 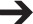 K, aa629 A 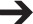 V	Cleavage and polyadenylation specificity factor subunit 3-i-like isoform (F: hydrolase activity)	AT1G61010, *CLEAVAGE AND POLYADENYLATION SPECIFICITY FACTOR 73-I* (*CPSF73-I*), protein involved in the mRNA polyadenylation in the nucleus
Bv9_227370_yfgr.t1 (yfgr)^a^^,^^b^^,^^c^	aa144 T 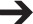 A, from aa191 changed sequence by a frame shift mutation, at aa204 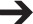 STOP codon resulting in a protein with 203 instead of 633aa	Cleavage and polyadenylation specificity factor subunit 3-i-like isoform (F: hydrolase activity)	AT1G61010, *CLEAVAGE AND POLYADENYLATION SPECIFICITY FACTOR 73-I* (*CPSF73-I*), protein involved in the mRNA polyadenylation in the nucleus
Bv9_227380_faqz.t1 (faqz)^a^^,^^b^^,^^c^	aa31 L 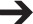 I	Probable plastidic glucose transporter 3 (C: trans-Golgi network, endosome, integral to membrane; P: negative regulation of biological process, carbohydrate transmembrane transport, determination of bilateral symmetry, xylem and phloem pattern formation, flower morphogenesis; F: sugar:hydrogen symporter activity)	AT1G79820, *SUPPRESSOR OF G PROTEIN BETA1* (*SGB1*), protein involved in the carbohydrate transport through membranes
**Non-coding genes based on RefBeet-1.1**	**P**_br_ **specific nucleotide changes compared to RefBeet-1.1 and P**_b_	**Homology to published miRNAs based on mirBase**	**Number of putative target genes based on RefBeet-1.1 gene models**
ncRNA_2240/MIR1122 (nc2240)^a^	No	*Brachypodium distachyon* miR5181d, involved in cold stress response; *Brachypodium distachyon* miR5174e stem-loop, involved in reprogramming leaf growth during drought stress	12 (non-related to flowering)

a *RNA evidence according to RNAseq and EST data of RefBeet-1.1 (Dohm et al., 2014)*.

b *RNA evidence according to RNAseq data derived from shoot apices of annual sugar beet accession 001684 during floral transition (Tränkner et al. unpublished)*.

c *RNA evidence according to SuperSAGE data derived from leaves of non-vernalized and vernalized KWS2320 sugar beets (Vogt et al. unpublished)*.

Furthermore, we investigated which of these genes are transcribed in leaves and shoot apices of beet. We screened two unpublished transcriptome data sets derived from shoot apices of an annual beet and from leaves of vernalized and non-vernalized plants of a biennial sugar beet. Yfgr.t1 is transcribed in shoot apices of the annual beet during floral transition and upregulated during vernalization in the biennial genotype (Figure S3), whereas transcripts of uapa.t1 and tswg.t1 were not detected. Thus, we excluded both of the non-expressed genes from being a *BR*_1_ candidate and assumed yfgr.t1 as the best candidate gene for *BR*_1_. For completeness, transcripts of iirc.t1/t2 were detectable at a very low level only in shoot apices of annual beets, whereas transcripts of ksuy.t1, dgic.t1, pgzt.t1, oeyr.t1, faqz.t1, and kzoy.t1 were clearly detectable in shoot apices of annual beets as well as leaves of biennial beets (Figure S3).

The *BR*_1_ candidate gene yfgr.t1 shares 81% identity at the protein level with the *A. thaliana CLEAVAGE AND POLYADENYLATION SPECIFICITY FACTOR 73-I* (*AtCPSF73-I*) gene. In contrast to *Arabidopsis, B. vulgaris* contains a second *CPSF73-I* homologous gene, oeyr.t1, which shows 83% identity with *AtCPSF73-I* at protein sequence level. Following we named yfgr.t1 *BvCPSF73-Ia* and oeyr.t1 *BvCPSF73-Ib*. *BvCPSF73-Ib* is also located on the *BR*_1_ locus, 954 bp upstream of *BvCPSF73-Ia* (Figure 2D, oeyr/yfgr). Both genes show a sequence identity of 97% at coding sequence and protein sequence level. Furthermore, both genes show a similar transcription pattern in annual and biennial sugar beets (Figure S3). Thus, we suggest that both genes originated from a common ancestor and possess a similar or same function.

## Discussion

### Post-winter bolting resistance is controlled by a major gene on chromosome 9

Post-winter bolting resistance is essential for prospective winter beet cultivars. While the bolting loci *BTC1* and *BvBBX19* control vernalization requirement, which distinguishes annual and biennial beets, the major QTL *BR*_1_ controls bolting after vernalization and distinguishes between biennial and never-bolting beets (Pfeiffer et al., 2014). Never-bolting is an absolute requirement to transform sugar beet from spring into a winter crop. In this study, we physically mapped the *BR*_1_ locus to a single region on chromosome 9. This region is located at RefBeet-1.1 scaffold Bvchr9.sca026 and includes 11 genes.

However, our F_2_ population showed a non-Mendelian segregation of the bolting-resistant phenotype with a clear over-representation of bolting plants. A natural selection of F_2_ plants against post-winter bolting resistance was excluded, because codominant markers of the *BR*_1_ locus segregated in a 1:2:1 ratio which is typical for F_2_ populations. Thus, additional loci might be expected. However, there was no evidence for another locus. Mapping-by-sequencing allows the detection of candidate sequence polymorphisms on a genome-wide level. Thereby, homozygosity is extended around the causal locus of the recessive trait due to genetic linkage (Schneeberger, 2014). In this study, we had detected chromosome 9 scaffolds 26, 25, and 24 which displayed the highest numbers of monomorphic positions per mega base pair. A considerably lower number or even no monomorphic positions were detected for all other scaffolds covering the whole genome of sugar beet. Scaffolds 24 and 25 are located upstream of scaffold 26 which is located in the telomeric region of chromosome 9 (Dohm et al., 2014). Both scaffolds had a clearly reduced number of candidate positions compared to scaffold 26. They point to scaffold 26 as the precise location of the *BR*_1_ gene due to the gradual enrichment of *br*_1_ alleles around the *BR*_1_ locus resulting in homozygosity at *BR*_1_. Previously, Pfeiffer et al. (2014) had detected only one QTL for post-winter bolting resistance using bolting rate data of 186 F_3_ families of the same population. The interval of the *BR*_1_ QTL completely covers the identified 103 kb *BR*_1_ region, showing a perfect co-localization. In conclusion, mapping-by-sequencing in combination with QTL mapping pointed exclusively to the *BR*_1_ locus as major locus controlling post-winter bolting resistance in beet.

### Incomplete penetrance of the bolting resistance phenotype

Surprisingly, 72.6% of the F_2_ plants that were homozygous for the *br*_1_ allele exhibited a bolting phenotype. F_3_ families derived from these plants after selfing showed bolting rates from 0 to 0.78 with an average of 0.27. Since the bolting-resistant phenotype of this mapping population was clearly linked to the recessive *br*_1_ allele, these findings suggest an incomplete penetrance of the *br*_1_ allele derived from the bolting-resistant accession BETA 1773. Incomplete penetrance of *br*_1_ was also implied through varying bolting rates of BETA 1773 in previous experiments. For example, Pfeiffer et al. (2014) reported bolting rates of 0.12 and 0.5 under field conditions in Germany. Under the synonyms “Kaweaa” or “Kleinwanzleben AA/Klein AA” (IPK, 2006; JKI, 2012), BETA 1773 showed bolting rates from 0.14 to 0.85 after over-winter cultivation in Spain (Lasa and Medina, 1978) and an average bolting rate of 0.57 after over-winter cultivation in England in 1971 that varied from 0.78 to 0.92 in 1972 depending on the sowing date in autumn. Progenies of bolting-resistant plants that had flowered after a second vernalization showed bolting rates between 0.63 and 0.77 after over-winter cultivation (Wood and Scott, 1975). Thus, a high variation for post-winter bolting resistance is observable within the original accession, its subsequent generations and within the crossing population used here.

Lasa and Medina (1978) determined for BETA 1773 a positive correlation between bolting and the number of days with minimum air temperatures between 3 and 10°C. In biennial beets, bolting is controlled by photothermal induction, requiring periods of cold followed by long-day conditions (Owen et al., 1940). Thereby, additional light units can replace temperature units (Steinberg and Garner, 1936; Stout, 1946). Photothermal induction is genotype-specific concerning vernalization temperature, duration of cold exposure and day length after cold-treatment. Temperatures from 2 to 10°C are mostly sufficient for thermal induction, whereby the highest bolting rates are obtained at about 4°C (Stout, 1946). Our climate chamber experiments confirmed that the bolting-resistant phenotype is strongly affected by photothermal induction. Two F_3_ families that were homozygous for the recessive *br*_1_ allele reached higher bolting rates after 4°C cold treatment and subsequent 22 h light exposure than under suboptimal bolting induction conditions at higher vernalization temperatures or with less light units. Since the bolting rates of both F_3_ families varied depending on the photoperiod as well as the vernalization temperature, *BR*_1_ seems to integrate signals from the photoperiodic and vernalization pathway assuming a functional position downstream of both flowering pathways. Additionally, the bolting-resistant phenotype might be also regulated by endogenous factors because F_3_ family 112206 showed in the climate chamber experiments higher bolting rates than 112210, whereas under 16 h artificial light both families were completely bolting-resistant.

The results of our study indicate incomplete penetrance of the bolting resistance phenotype. Incomplete penetrance is a widespread feature which appears in a wide range of organisms affecting diverse traits. For example, a major locus controlling *in situ* gynogenesis of maize shows incomplete penetrance (Barret et al., 2008). In tomato, mutants of the *TERMINATING FLOWER* (*TMF*) gene exhibit a single-flower phenotype instead of multi-flowered inflorescences with 50% penetrance in the original mutant background and varying levels up to 100% in the 3rd backcross generation (MacAlister et al., 2012). Thereby, *tmf* mutants developed solitary flowers through precocious activation of a floral specification complex. The authors proposed the presence of unknown modifiers to explain the varying penetrance levels, but they did not give further information about the modifier's type or mode of action. Reasons for incomplete penetrance are diverse and different regulatory mechanisms are involved. As reviewed by Cooper et al. (2013), the penetrance of a phenotype can be affected by the specific mutation itself, copy number variations, or differential allelic expression. Furthermore, unlinked genes, environmental or developmental (age-dependent) factors or epigenetic regulation of expression can affect the penetrance of a phenotype. The role of DNA methylation in bolting has been described on a whole genome scale (Trap-Gentil et al., 2011; Hébrard et al., 2016). Thus, conditional epigenetic modification of the *BR*_1_ locus could result in altered gene activities giving rise to a non-Mendelian segregation of the bolting phenotype. In addition, mutations in genes which act redundantly can result in phenotypes with incomplete penetrance as described for the auxin influx carrier genes *aux1, lax1*, and *lax2* of *Arabidopsis*. The *aux1, lax1*, and *lax2* single mutants and *aux1 lax2* and *lax1 lax2* double mutants showed no obvious defects during embryo development, whereas *aux1 lax1* double mutants and triple mutants showed incomplete penetrance of 4–22.1%. Thereby the penetrance level and severity of defects was increased in triple mutants, clearly demonstrating a functional redundancy of these auxin influx carrier genes (Robert et al., 2015). The regulatory mechanism that controls penetrance of the bolting resistance phenotype of sugar beet is unknown until now.

### Indications that a cleavage and poly-adenylation specificity factor 73-I homolog underlies the *BR_1_* phenotype

Out of the 11 genes located within the physically mapped *BR*_1_ interval, we propose yfgr.t1 (*BvCPSF73-Ia*) as *BR*_1_ candidate. *BvCPSF73-Ia* fulfilled all criteria that we expected from a hypothetical *BR*_1_ gene: (i) It is located at the *BR*_1_ locus, (ii) it is transcribed during floral transition in leaves and shoot apices of regularly bolting beet genotypes, (iii) the BETA 1773 allele encodes a severely truncated protein because of a BETA 1773-specific 2 bp deletion within the coding sequence, and (iv) a loss-of-function can be assumed. *BvCPSF73-Ia* is a homolog of the *Arabidopsis* gene *CPSF73-I*. CPSF proteins are essential for the polyadenylation of mRNA and splicing of terminal introns in eukaryotes. In *Arabidopsis*, the CPSF proteins CPSF160, CPSF100, CPSF73, and CPSF30 form a complex which recognizes and directly binds to polyadenylation sites of pre-mRNAs. After recruitment of further factors, the cleavage endonuclease CPSF73 removes the RNA 3' end and the synthesis of the poly(A) tail follows (Shi and Manley, 2015). The *Arabidopsis* genome contains 5 *CPSF* genes including two *CPSF73* homologs, *AtCPSF73-I* and *AtCPSF73-II*. Both *AtCPSF73* genes are essential for plant development because knockout and knockdown mutants are lethal (Xu et al., 2004, 2006). Mutations within 3′-end processing factors can affect flowering through alternative polyadenylation of mRNAs or deficient mRNA processing which induces gene silencing (Herr et al., 2006; Zhang et al., 2015). Previous studies showed also *in vivo* associations between AtCPSF proteins and the FY protein. FY controls floral transition through down-regulation of the floral repressor gene *FLC*, which is a major regulator of vernalization requirement (Simpson et al., 2003; Manzano et al., 2009). If *BvCPSF73-Ia* has a similar function like its *Arabidopsis* homolog, a loss-of-function of *BvCPSF73-Ia* can result in bolting resistance after vernalization.

Interestingly, sugar beet contains three *CPSF73* homologs (gene models yfgr.t1, oeyr.t1, and gfgf.t1). While *BvCPSF73-Ia* (yfgr.t1) and *BvCPSF73-Ib* (oeyr.t1) show highest identity to *AtCPSF73-I*, gfgf.t1 is the homolog of *AtCPSF73-II*. *BvCPSF73-Ia* and *BvCPSF73-Ib* are located next to each other within the *BR*_1_ locus. The high sequence identity between both *BvCPSF73-I* homologs suggests an ancient gene duplication. Due to the same origin and the similar transcription pattern during floral transition, we assume that *BvCPSF73-Ia* and *BvCPSF73-Ib* have a similar function and might act redundantly. Accordingly, we hypothesize that beets with null alleles of *BvCPSF73-Ia* will express intact BvCPSF73-I protein due to *BvCPSF73-Ib*, but that the total BvCPSF73-I protein amount will be lower than in wild-type plants. However, the expression level of BvCPSF73-I might be crucial for plant development because the overexpression, knockout, or knockdown of *AtCPSF73-I* in *Arabidopsis* is lethal, whereas a slightly increased expression results in normal looking but male sterile plants (Xu et al., 2006). Based on our hypothesis, bolting resistance might be caused when the concentration of BvCPSF73-I protein falls below a certain threshold. In contrast, the incomplete phenotype of BETA 1773 plants can result from an environment-dependent or genotype-specific higher expression of oeyr.t1. Otherwise, incomplete penetrance might be the result of a threshold-specific expression of a putative *BvCPSF73-I* targeted flowering gene like the floral promoter *BvFT2*.

Although *BvCPSF73-Ia* is the most promising candidate gene, other genes of the *BR*_1_ locus cannot be fully excluded. For example, the putative bZIP transcription factor kzoy.t1 contains a P_*br*_ specific 7 bp deletion in the promoter region that might affect post-winter bolting behavior by altered gene expression levels. Thus, further research is necessary to ultimately identify the *BR*_1_ gene and to unravel the molecular mechanism that leads to post-winter bolting resistance. Understanding the mechanism of incomplete penetrance of *BR*_1_-mediated post-winter bolting resistance will allow identifying *BR*_1_ modifier genes that can subsequently be used for winter beet breeding. Thus, functional analysis of the *BR*_1_ gene like spatial and temporal expression analysis, complementation of *BR*_1_ homozygous plants, knockout or knockdown of functional alleles by mutagenesis or targeted genome editing must follow. Moreover, the genes targeted by *BR*_1_ must be identified. If yfgr.t1 underlies the *BR*_1_ locus, then targeted genes might have altered polyadenylation sites or they show a reduced expression through posttranscriptional regulation. If kzoy.t1 is the *BR*_1_ gene, then homozygous dominant and homozygous recessive plants will show different transcript levels of target genes that can be identified by RNAseq approaches.

### Relevance for breeding winter beet

Due to the monogenic inheritance of the *BR*_1_-mediated post-winter bolting resistance, post-winter bolting resistance of BETA 1773 can easily be introgressed into winter beet genotypes. Thereby the *BR*_1_-specific InDel marker CAU3903 or a marker specific for the 2 bp deletion in yfgr.t1 will allow the selection of progenies with the recessive *br*_1_ allele. Modern sugar beet cultivars are mostly hybrids. Thus, the introgression of the recessive *br*_1_ allele into both parental lines is necessary to obtain homozygous recessive hybrids. Since *BR*_1_-mediated post-winter bolting resistance shows incomplete penetrance, further loci for bolting resistance or bolting delay must be combined in a genotype to acquire complete bolting resistance after winter. For this, the natural variation of post-winter bolting resistance in the *B. vulgaris* gene pool must be explored further. Furthermore, the *Beta* gene pool should be scanned for *BR*_1_ haplotypes to identify *br*_1_ alleles with increased penetrance. However, haplotypes causing complete bolting resistance will be rare because bolting-resistant beets cannot be multiplied and were lost during previous breeding and propagation processes. Simultaneously to the identification and pyramiding of bolting resistance alleles, systems have to be developed that allow controlled bolting induction of bolting-resistant beets for seed production.

## Author contributions

CT supervised the project, designed and performed experiments and wrote the manuscript. IL and IG designed and performed computational analyses and wrote the manuscript. MS performed NGS. NE, NP, ST and FK provided phenotypic and genotypic data. SV and AM provided SuperSAGE-data. CJ supervised the experiments and revised the manuscript. All authors read and approved the final manuscript.

## Funding

This study was funded by the DFG Priority Program SPP1530 grant numbers TR1088/1-1 and GR 3526/2-1, and the German Federal Ministry of Education and Research (BMBF) program grant numbers FKZ 0315465B and 0315058A.

### Conflict of interest statement

The authors declare that the research was conducted in the absence of any commercial or financial relationships that could be construed as a potential conflict of interest.
