# Supplementary material for: A Detailed Analysis of the BR1 Locus Suggests a New Mechanism for Bolting after Winter in Sugar Beet (Beta vulgaris L.)
Source: Front Plant Sci. 2016 Nov 14;7:1662. doi: 10.3389/fpls.2016.01662 (PMC5107561; doi:10.3389/fpls.2016.01662)
Supplement: Supplementary file 1 [file DataSheet1.docx]

# Supplementary Material

Table S1. Summary of processed whole genome sequencing data.

| **Bulk/ pool** | **DNA-Library** | **No. of plants** | **Total number of reads (101bp)** | **Number of reads after quality control** | **Percent-age of mapped reads % (Bowtie2)** | **Percent-age of uniquely mapped reads %** | **Total cover-agea of uniquely mapped reads** |
| --- | --- | --- | --- | --- | --- | --- | --- |
| **Bolting-resistant** | B0679 | 6 | 319,698,534 | 264,237,494 | 98.23 | 31.05 | 14 |
|  | B0680 | 6 | 353,460,556 | 291,137,892 | 98.25 | 31.81 | 16 |
|  | B0681 | 7 | 361,224,474 | 297,612,542 | 98.33 | 32.57 | 17 |
|  | B0682 | 7 | 545,946,848 | 451,136,222 | 98.26 | 32.07 | 25 |
|  | **in total** | **26** | **1,580,330,412** | **1,304,124,150** | **98.27** | **31.88** | **72** |
| **Bolting** | B0683 | 148 | 352,074,774 | 294,113,744 | 98.10 | 32.41 | 17 |
|  | B0684 | 149 | 348,801,012 | 296,497,012 | 98.01 | 32.34 | 17 |
|  | **in total** | **297** | **700,875,786** | **590,610,756** | **98.06** | **32.38** | **34** |

aconsidering the reference genome size of RefBeet-1.1 of 569.0Mbp

Table S2. List of markers including primer sequences.

| Marker | Position in RefBeet-1.1 Bvchr9.sca026 | Poly-morphism | Forward primer | Reverse primer | Detection | PCR product BETA 1773 | PCR product 93161P |
| --- | --- | --- | --- | --- | --- | --- | --- |
| CAU3839a | 6562842..6562969 | 18 bp InDel | CATTATGGAACGGAGGAAG | CCCCTGTAGGTCTTTAAG | PCR: 94°C, 2' + [(94°, 30'' + 55°C, 30'' + 72°C, 30'') x 34] + 72°C, 5'  GE: 3% agarose, 110V, 60' | 110 bp | 128 bp |
| CAU3841a | 4245162..4245800 | 5 bp InDel | ACCCGAGAGCACAACAACTTAT | TTTATTTTCTTGTGTAGTGGACCAT | PCR: 94°C, 2' + [(94°, 30'' + 59°C, 30'' + 72°C, 30'') x 34] + 72°C, 5'  GE: 3% agarose, 110V, 60' | 639 bp | 644 bp |
| CAU3888b | 5443916..5444109 | 27 bp InDel | GAAAAGAGAGCCACTTCCATG | GCAATGGAGGAACAATCTGTG | PCR: 94°C, 2' + [(94°, 30'' + 59°C, 30'' + 72°C, 30'') x 34] + 72°C, 5'  GE: 3% agarose, 110V, 50' | 167 bp | 194 bp |
| CAU3892b | 3071626..3071784 | 17 bp InDel | CTACACATRACCCCATTATYTAC | GTCAATTCRGAGKTCAGTTATG | PCR: 94°C, 2' + [(94°, 30'' + 58°C, 30'' + 72°C, 30'') x 34] + 72°C, 5'  GE: 3% agarose, 110V, 50' | 142 bp | 159 bp |
| CAU3902b | 4578380..4578507 | 11 bp InDel | GATCTGGGCTCACTTATGATG | GGATGCAAGATGTCTTCCTAC | PCR: 94°C, 2' + [(94°, 30'' + 60°C, 30'' + 72°C, 30'') x 34] + 72°C, 5'  GE: 3% agarose, 110V, 60' | 117 bp | 128 bp |
| CAU3903b | 5089089..5089296 | 9 bp InDel | GTGATTATAATTGAACATATACCTG | GAGTTGTAAGTCATGAGGCAC | PCR: 94°C, 2' + [(94°, 30'' + 58°C, 30'' + 72°C, 30'') x 34] + 72°C, 5'  GE: 3% agarose, 110V, 60' | 208 bp | 199 bp |
| CAU3904b | 5098777..5098989 | 8 bp InDel | CAGACAACTCATGTAATCGTC | CATTAAATCAAAAAAGAGGGAC | PCR: 94°C, 2' + [(94°, 30'' + 56°C, 30'' + 72°C, 30'') x 34] + 72°C, 5'  GE: 3% agarose, 110V, 60' | 213 bp | 221 bp |
| CAU3905b | 5609969..5610101 | 11 bp InDel | GGTCTTAGTCAAATTGTCTTTC | GCTTGCTTTCATCAATTCATG | PCR: 94°C, 2' + [(94°, 30'' + 56°C, 30'' + 72°C, 30'') x 34] + 72°C, 5'  GE: 3% agarose, 110V, 60' | 144 bp | 133 bp |
| CAU3906b | 4749496..4749650 | 19 bp InDel | CTCATTGTGGCTCGACGAAAC | CAGTTATATATTGATCACGATGCAC | PCR: 94°C, 2' + [(94°, 30'' + 61°C, 30'' + 72°C, 30'') x 34] + 72°C, 5'  GE: 3% agarose, 110V, 50' | 156 bp | 175 bp |
| CAU3907b | 4349760..4349915 | 8 bp InDel | CTAGATAGTCCGTAGATATTG | CTGGTACTTTCCCACTAGAAC | PCR: 94°C, 2' + [(94°, 30'' + 56°C, 30'' + 72°C, 30'') x 34] + 72°C, 5'  GE: 3% agarose, 110V, 60' | 164 bp | 156 bp |
| CAU4062b | 4925225..4925425 | SNP, Y026_SNPs 39C/T, 56T/C, 91T/C | Y025: GGTGTTAAGATGATACATAATAAATC | Y026: CATAGAAGGATGTGAAGGAGAC | PCR: 94°C, 2' + [(94°, 30'' + 58°C, 30'' + 72°C, 1:30) x 34] + 72°C, 5'  Sanger sequencing with Y025 | 201 bp (39C, 56T, 91T) | 201 bp (39T, 56C, 91C) |

amarker used by Pfeiffer et al. 2014, bmarker developed in this study, PCR polymerase chain reaction, GE gel electrophoresis

Table S3. Crossover events in bolting-resistant F2 plants 313 and 297 define the *BR1* locus interval. A = homozygous for the BETA1773 allele, B = homozygous for the 93161P allele, H = heterozygous, U = unknown.

| Crossing parents and bolting-resistant F2 plants | NGS library number | CAU  3892 | CAU  3841 | CAU  3907 | CAU  3902 | CAU  3906 | CAU  4062 | CAU  3903  *BR1* locus | CAU  3904 | CAU  3888 | CAU  3905 |
| --- | --- | --- | --- | --- | --- | --- | --- | --- | --- | --- | --- |
| 080299_105 (BETA 1773, seed plant) | no | A | A | A | A | A | A | A | A | A | A |
| 93161P (pollinator) | no | B | B | B | B | B | B | B | B | B | B |
| 102946_034 | B0679 | A | A | A | A | A | A | A | A | A | A |
| 102946_076 | B0679 | H | A | A | A | A | A | A | A | A | A |
| 102946_079 | B0680 | H | A | A | A | A | A | A | A | A | A |
| 102946_102 | B0679 | A | A | A | A | A | A | A | A | A | A |
| 102946_109 | B0681 | A | A | A | A | A | A | A | A | A | A |
| 102946_134 | B0681 | A | A | A | A | A | A | A | A | A | A |
| 102946_149 | B0681 | A | A | A | A | A | A | A | A | A | A |
| 102946_158 | B0681 | A | A | A | A | A | A | A | A | A | A |
| 102946_166 | B0681 | A | A | A | A | A | A | A | A | A | A |
| 102946_216 | B0680 | H | A | A | A | A | A | A | A | A | A |
| 102946_221 | B0682 | A | A | A | A | A | A | A | A | A | A |
| 102946_236 | B0680 | A | A | A | A | A | A | A | A | A | H |
| 102946_241 | B0680 | A | A | A | A | A | A | A | A | A | A |
| 102946_293 | B0680 | A | A | A | A | A | A | A | A | A | A |
| 102946_297 | B0679 | A | A | A | A | A | A | A | H | H | H |
| 102946_313 | B0682 | H | H | H | H | H | H | A | U | A | A |
| 102946_314 | B0682 | A | A | A | A | A | A | A | A | A | A |
| 102946_317 | B0680 | A | A | A | A | A | A | A | A | A | A |
| 102946_338 | B0682 | A | A | A | A | A | A | A | A | A | A |
| 102946_357 | B0682 | A | A | A | A | A | A | A | A | A | A |
| 102946_358 | B0681 | A | A | A | A | A | A | A | A | A | A |
| 102946_365 | B0682 | A | A | A | A | A | A | A | A | A | A |
| 102946_393 | B0681 | A | A | A | A | A | A | A | A | A | A |
| 102946_398 | B0679 | A | A | A | A | A | A | A | A | A | A |
| 102946_406 | B0682 | A | A | A | A | A | A | A | A | A | A |
| 102946_409 | B0679 | A | A | A | A | A | A | A | A | A | A |

**Table S4****.** Phenotypic analysis of 410 F2 plants with different *BR1* genotypes as determined by marker CAU3903.The *br1* allele was derived from the bolting-resistant sugar beet BETA 1773, the *BR1* allele from the bolting sugar beet 93161P.

| F2 phenotypes after cold-treatment | Number of F2 plants | *br1br1*  BETA 1773 allele homozygous | *BR1br1*  heterozygous | *BR1BR1*  93161P allele homozygous |
| --- | --- | --- | --- | --- |
| Boltinga | 384 | 69 | 201 | 114 |
| Bolting-resistant | 26 | 26 | 0 | 0 |
| In total | 410 | 95 | 201 | 114 |
| 2 for H0 = 1:2:1 (segregation of codominant markers in F2 populations) | | 1.917ns  H0 accepted | | |

aLate and incomplete bolting plants are classified as bolting, nsnot significant at tab2 = 5.99 (df = 2, α = 0.05)


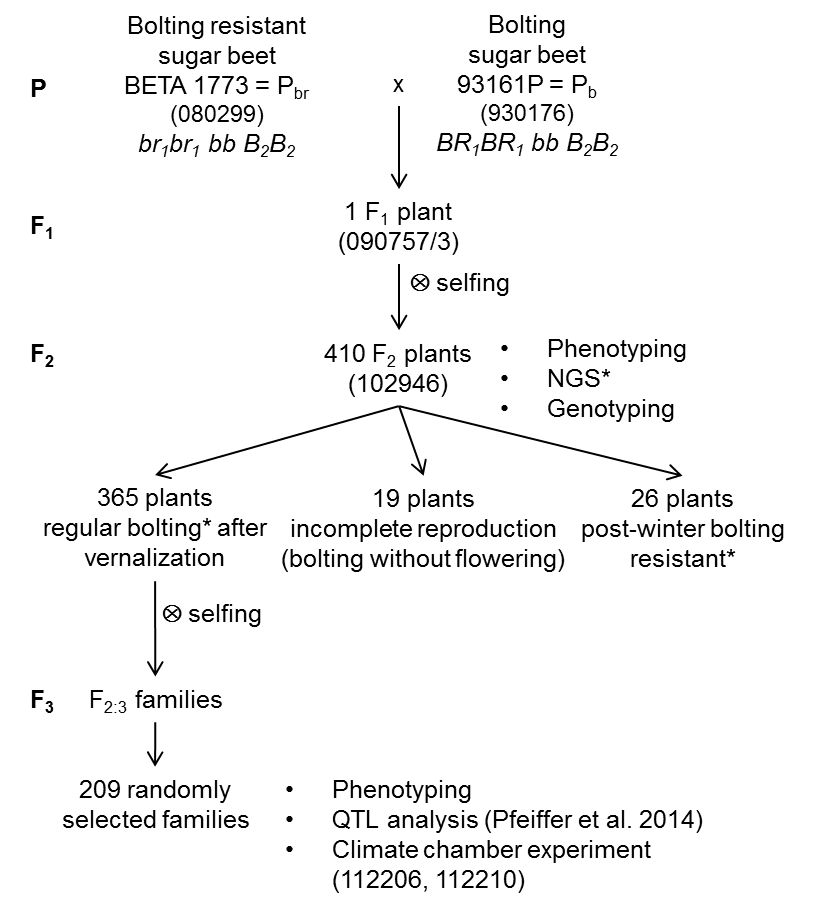


**Figure S1.** Crossing scheme for the development of the F2 and F3 mapping populations that segregated for bolting behavior after cold treatment. The F2 offspring was derived from a single F1 plant of a cross between sugar beet accessions BETA 1773 and 93161P. Both parents were homozygous for the biennial *btc1* allele (*B* locus) and the dominant *BvBBX19* allele (*B2* locus). The F3 progenies were derived by selfing F2 plants. Plant accession codes are given in brackets.

## Bv9_227290_ksuy.t1

ksuy.t1_BETA1773 MASIIANISLPLVINGAKPSLIPTSNFPVCVFPVSERRGRLAVVVRATGDSSDSSGSLSV

ksuy.t1_93161P MASIIANISLPLVINGAKPSLIPTSNFPVCVFPVSERRGRLAVVVRATGDSSDSSGSLSV

ksuy.t1_RefBeet-1.1 MASIIANISLPLVINGAKPYLIPTSNFPVCVFPVSERRGRLAVVVGATGDSSDSSGSLSV

******************* ************************* **************

ksuy.t1_BETA1773 VESVQNLWDNGEDRIAVIGLGLTAVIALWASLNLVAAIDKLPVIPGFLEFVGILFSSWFV

ksuy.t1_93161P VESVQNLWDNGEDRIAVIGLGLTAVIALWASLNLVAAIDKLPVIPGFLEFVGILFSSWFV

ksuy.t1_RefBeet-1.1 VESVQNLWDNGEDRIAVIGLGLTAVIAFWASLNLVAAIDKLPVIPGFLEFVGILFSSWFV

***************************:********************************

ksuy.t1_BETA1773 YRYLLFKPDRQELFRNMNKSISNILGQ*

ksuy.t1_93161P YRYLLFKPDRQELFRNMNKSISNILGQ*

ksuy.t1_RefBeet-1.1 YRYLLFKPDRQELFRNMNKSISNILGQ*

****************************

## Bv9_227300_uapa.t1

uapa.t1_BETA1773 MESSASSPS-----PSSPIYTPSSPSYSPPPSPEYSPESPSYTPSTPEYSPQSYKDYLHD

uapa.t1_93161P MESSASSPS-----PSSPVYTPSSPSYSPPPSPEYSPESPSYTPSTPEYSPQSYKHYLHD

uapa.t1_RefBeet-1.1 MESSASSPSSPQYSPSSPIYTPSSPSYSPPPSPEYSPESPSYTPSTPEYSAQSYKHYLHD

********* ****:******************************* ****.****

uapa.t1_BETA1773 SPQPYMSTEYSPQSPSYTPSSPVYTPESPEYSSQSPFYTPLSPEYSPQSPFYTPVQSPEY

uapa.t1_93161P SPQPYMSTEFSPQSPSYTPSSPVYTPESPEYSSQSPFYTPLSPEYSPQSPFYTPVQSPEY

uapa.t1_RefBeet-1.1 SPQPYMSTEYSPQSPSYTPCSPVYTPESPEYSSQSPFYTPLSPEYSPQSPFYTPVQSPEY

*********:*********.****************************************

uapa.t1_BETA1773 TPESPEYTPQSPYYTPLSPEYTPQSPKYTPESPEYTPESPEYTPQSPEYTPQSPYYTPSS

uapa.t1_93161P TPETPEYTPQSPYYTPLSPEYTPQSPKYTPESPEYTPESPEYTPQSPEYTPQSPYYTPSS

uapa.t1_RefBeet-1.1 TPETPEYTPQSPYYTPLSPEYTPQSPKYTPESPEYTPESPEYTPQSPEYTPQSPYYTPSS

***:********************************************************

uapa.t1_BETA1773 PEYTPQSPEYTPESPEYTPQSPYYTPLSPEYSAGSWTWSSSYTPVCCI*-------

uapa.t1_93161P PEYTPQSPEYTPESPEYTPQSPYYTPLSPEYSTGSWTWSSSYTPVCCI*-------

uapa.t1_RefBeet-1.1 PEYTPQSPEYTPESPEYTPQSPYYTPLSPEYSTGSWTWSSSYTPVCCLLYLIKDD*

********************************:**************:

## Bv9_227310_tswg.t1

tswg.t1_BETA1773 MRPPQPYNYHNNHHYHHRNYHTRHSKQPPLLPTPPQYRHSPPFGFHQAPPLPPISRTYYH

tswg.t1_93161P MRPPQPYNYHNNHHYHHRNYHTRHSKQPPLLPTPPQYRHSPPFGFHQSPPLPPISRTYYH

tswg.t1_RefBeet-1.1 MRPPQPYNYHSNHHYHHRNYHTRHSKQPPLLPTPPQYRHSPPFGFHQAPPLPPISPTYYH

********** ************************************ ******* ****

tswg.t1_BETA1773 HQPPLLIPKYKNVNISPLPLDHHKLVSPPPAELRVYSPSSPFYSSVSPNYCGNYYYPHTT

tswg.t1_93161P HQPSLLIPKYKNVYISPLPLDHHTLVSPPPAELRVYSPSSPFYSSVSPNYCGNYYYPHTT

tswg.t1_RefBeet-1.1 HQPPLLIPKYKNVNISPLPLDHHTLVSPPPAELRVYSPSSPFYSSVSPNYCGNYYYPHTT

*** ********* ********* ************************************

tswg.t1_BETA1773 SDNNEYD-ADYYSPWSPKYSLPSPVYLPPSPSSSSSSLDQTKQPSESPDDSPPSSPVYSP

tswg.t1_93161P IDNNEYDHADYYSPWSPKYSLPSPVYLPPSPSSSSS-LDQTKQPSESPDDSPPSSPVYSP

tswg.t1_RefBeet-1.1 SDNNEYDHADYYSPWSPKYSLPSPVYLPPSPSSSS--LDQTKQPSESPDDSPPSSPVYSP

****** *************************** ***********************

tswg.t1_BETA1773 SSSLLPPS-----------L---F*

tswg.t1_93161P SSSPLPPSFDHSLPYSPSYVPYTP*

tswg.t1_RefBeet-1.1 SSSPLPPSFDHSLPYSPSYVPYTP*

*** **** *

## Bv9_227320_iirc.t1/t2

iirc.t1_BETA1773 MVAKLIVSSSSRFLPFARALSTRPAFSLLTNVAYHTFLGKSAVNSAVQQQQQRNYTANYT

iirc.t1_93161P MVAKLIVSSSSRFLPFARALSTKPAFSLLTNVAYHTFLGKSALNSAVQQQQQRNYTANYT

iirc.t1_RefBeet-1.1 MVAKLIVSSSSRFLPFARALSTRPAFSLLTNVAYHTFLGKSAVNSAVQQQQQRNYTANYT

iirc.t2_BETA1773 MVAKLIVSSSSRFLPFARALSTRPAFSLLTNVAYHTFLGKSAVNSAVQQQQQRNYTANYT

iirc.t2_93161P MVAKLIVSSSSRFLPFARALSTKPAFSLLTNVAYHTFLGKSALNSAVQQQQQRNYTANYT

iirc.t2_RefBeet-1.1 MVAKLIVSSSSRFLPFARALSTRPAFSLLTNVAYHTFLGKSAVNSAVQQQQQRNYTANYT

********************** ******************* *****************

iirc.t1_BETA1773 RNLPSIVCKDLNKKSHQDFSFNLAKRCLVDGLNSTSQKNLLCSPVSTSAIINMLVPGSKG

iirc.t1_93161P RNLPSIVCKDLNKKSHQDFSFNLAKRCLVDGLHSTSQKNLLCSPVSTSAIINMLVPGSKG

iirc.t1_RefBeet-1.1 RNLPSIVCKDLNKKSHQDFSFNLAKRCLVDGLNSTSQKNLLCSPVSTSAIINMLVPGSKG

iirc.t2_BETA1773 RNLPSIVCKDLNKKSHQDFSFNLAKRCLVDGLNSTSQKNLLCSPVSTSAIINMLVPGSKG

iirc.t2_93161P RNLPSIVCKDLNKKSHQDFSFNLAKRCLVDGLHSTSQKNLLCSPVSTSAIINMLVPGSKG

iirc.t2_RefBeet-1.1 RNLPSIVCKDLNKKSHQDFSFNLAKRCLVDGLNSTSQKNLLCSPVSTSAIINMLVPGSKG

******************************** ***************************

iirc.t1_BETA1773 ETLYQFLDLLGLEEEKLNKSAMRLIDVVKPLNNVGGAPKIAFSNSIWLDQQYQLTQSYKE

iirc.t1_93161P ETLYQFLDLLGLEEEKLNKSAMRLIDAVKPLNNVGGAPKIAFSNSVWLDQQYQLTQSYKE

iirc.t1_RefBeet-1.1 ETLDQFLDLLGFEEEKLNKSAMRLIDVVKPLNNVGGAPKIAFSNSIWLDQQYQLTQSYKE

iirc.t2_BETA1773 ETLYQFLDLLGLEEEKLNKSAMRLIDVVKPLNNVGGAPKIAFSNSIWLDQQYQLTQSYKE

iirc.t2_93161P ETLYQFLDLLGLEEEKLNKSAMRLIDAVKPLNNVGGAPKIAFSNSVWLDQQYQLTQSYKE

iirc.t2_RefBeet-1.1 ETLDQFLDLLGFEEEKLNKSAMRLIDVVKPLNNVGGAPKIAFSNSIWLDQQYQLTQSYKE

*** ******* ************** ****************** **************

iirc.t1_BETA1773 LIKNVHNADVKSVDFLYQADDVMQEVNRWSRTKTEGMIKSILPETSYYNDTVMVLANALH

iirc.t1_93161P MIKNVHNADVKSVDFLYQADDVMEEVNRWSRRKTEGMIKSILPETSYYNDTVMVLANALH

iirc.t1_RefBeet-1.1 LIKNVHNADVKSVDFLYQADDVMEEVNRWSRTKTEGMIKSILPETSYYNDTVMVLANALH

iirc.t2_BETA1773 LIKNVHNADVKSVDFLYQADDVMQEVNRWSRTKTEGMIKSILPETSYYNDTVMVLANALH

iirc.t2_93161P MIKNVHNADVKSVDFLYQADDVMEEVNRWSRRKTEGMIKSILPETSYYNDTVMVLANALH

iirc.t2_RefBeet-1.1 LIKNVHNADVKSVDFLYQADDVMEEVNRWSRTKTEGMIKSILPETSYYNDTVMVLANALH

********************** ******* ****************************

iirc.t1_BETA1773 FKLGSSDETVDSNEELMYYCGCFDGSEVLRMPYKQSNTTVVDQDPRSFSMYVLVPSNPSG

iirc.t1_93161P FKLGSSDETVDSNEALMYYCGCFDGSEVLRMPYKQSNTTVVDQDPRSFSMYVLVPSNQSR

iirc.t1_RefBeet-1.1 FKLGSSDATVDSNEALMYYCGCFDGSEVLRMPYKQSNTTVVDQDPRSFSMYVLVPSNPSE

iirc.t2_BETA1773 FKLGSSDETVDSNEELMYYCGCFDGSEVLRMPYKQSNTTVVDQDPRSFSMYVLVPSNPSG

iirc.t2_93161P FKLGSSDETVDSNEALMYYCGCFDGSEVLRMPYKQSNTTVVDQDPRSFSMYVLVPSNQSR

iirc.t2_RefBeet-1.1 FKLGSSDATVDSNEALMYYCGCFDGSEVLRMPYKQSNTTVVDQDPRSFSMYVLVPSNPSE

******* ****** ****************************************** *

iirc.t1_BETA1773 ENDDPRWGVTPIKQKKSLREIIEEMKVDGKTVFEKQIKSQKVEISDILIPKFNLESEISL

iirc.t1_93161P ENDDPCWGVTPIKQKKSHQEIIEEMKVDGKTAFEKQIKRQMVEISDILVPKFNLESEISL

iirc.t1_RefBeet-1.1 ENDDPRWGVTPIKQKKSLQEIIEEMKVDGKTAFEKQIKRQKVEISDILIPKFNLESEISL

iirc.t2_BETA1773 ENDDPRWGVTPIKQKKSLREIIEEMKVDGKTVFEKQIKSQKVEISDILIPKFNLE-----

iirc.t2_93161P ENDDPCWGVTPIKQKKSHQEIIEEMKVDGKTAFEKQIKRQMVEISDILVPKFNLE-----

iirc.t2_RefBeet-1.1 ENDDPRWGVTPIKQKKSLQEIIEEMKVDGKTAFEKQIKRQKVEISDILIPKFNLE-----

***** *********** ************ ****** ********* ******

iirc.t1_BETA1773 SSTMNDLGLTFPFKAGQNELMRFVDSPISDLLYVTDITQKSRIECNNGGISFSVVTYTTL

iirc.t1_93161P SSTMNDLGLTFPFKAGQNELMRFIDSPISDLLYVTDITQKSRIECNKGGLSFSVVTYTTL

iirc.t1_RefBeet-1.1 SNTMNDLGLTFPFKAGQNELMRFIDSPISDLLYVTDITQKSRIECNKGGISFSVVTYTTL

iirc.t2_BETA1773 -----------------------------NLLYVTDITQKSRIECNNGGISFSVVTYTTL

iirc.t2_93161P -----------------------------NLLYVTDITQKSRIECNKGGLSFSVVTYTTL

iirc.t2_RefBeet-1.1 -----------------------------NLLYVTDITQKSRIECNKGGISFSVVTYTTL

**************** ** **********

iirc.t1_BETA1773 CGPQHWFPTSSKLKFVAENPFMFMIQEDISGAVISVGTMLSTPLSKS*

iirc.t1_93161P CGPQHWFPTSSKLKFVAENPFMFMIQEDISGAVVSVGTMLSTPLSKF*

iirc.t1_RefBeet-1.1 CGPQHWFPTSSKLKFVAENPFMFMIQEDISGAVVSVGTMLSTPLSKF*

iirc.t2_BETA1773 CGPQHWFPTSSKLKFVAENPFMFMIQEDISGAVISVGTMLSTPLSKS*

iirc.t2_93161P CGPQHWFPTSSKLKFVAENPFMFMIQEDISGAVVSVGTMLSTPLSKF*

iirc.t2_RefBeet-1.1 CGPQHWFPTSSKLKFVAENPFMFMIQEDISGAVVSVGTMLSTPLSKF*

********************************* ************ *

## Bv9_227330_dgic.t1

dgic.t1_BETA1773 MDWWGGKVSLGNLDFSGAVNKLQESVKSIEKNFDSALGLEDKLQSSDEASGPQTTSSSRN

dgic.t1_93161P MDWWGGKVSLGNLDFSGAVNKLQGSVKSIEKNFDSALGLEDKLQSSDEASGPQTTSSSRN

dgic.t1_RefBeet-1.1 MDWWGGKVSLGNLDFSGAVNKLQESVKSIEKNFDSALGLEDKLQSSDEASGPPTSSSSRN

*********************** **************************** * *****

dgic.t1_BETA1773 ALFDPVMAFMGNKEEDNVDESKEREIPQDEARSAGNKDDSSEHLESMQGQSVLEEKKVDG

dgic.t1_93161P ALFDPVMAFMGNKEEDNVDESKEREIPQDEARSAENKDYSSEHLESMQGQSVVEEKKVDG

dgic.t1_RefBeet-1.1 ALFDPVMAFMGNKEEDNVDESKEREIPQDEARIAENKDDSSEHLESMQGQSVVEEKKVDG

******************************** * *** ************* *******

dgic.t1_BETA1773 ADTEVQHVIEPVLSPEEEEGGVKEQMDYVASDAVDEVVTMTEDSGEVRSDAQESTASPEV

dgic.t1_93161P ADTEVQHVIEPVLSPEEEEGGVKEQMDYVASEAVDEVVTMIEDSGEVRSDAQESTASPEV

dgic.t1_RefBeet-1.1 VDTVVQHVIEPVLSPQEEEGGVKDQMDYVASEAVDEVDTMTEYSSEVRSDAQESTASPEV

** *********** ******* ******* ***** ** * * ***************

dgic.t1_BETA1773 TIQDAQETSKQLDGEGEPEEGSLEHSESAESKVVDDQVNQHHSDNFINREASDAVEPLES

dgic.t1_93161P TIQDAQETSKQLDGEGESEEGSLEHSESAESKVVDDQVNQHHTDNFINREASDAVEPLES

dgic.t1_RefBeet-1.1 TIQDAQETSKQLDGEEKPEEGSLEHSESAESKVVDDQANQHHTDNFITREASDAVEPLES

*************** ******************* **** **** ************

dgic.t1_BETA1773 TQELAVGEGTPNQEEVSSDSQAGNLIEHSENVVGIADAEIESSTGSVNINNSSSPEFLTN

dgic.t1_93161P TQELAVGGGTPKQEEVSSDSQAGNLVEHSENGVGIADAEIESSTGSVNINNSSSSEFLTN

dgic.t1_RefBeet-1.1 TQELAVGGGTPKQEEVSSDSQAGNLVEHSENVVGIADAEIESSTGSVNINNSSSPEFLTN

******* *** ************* ***** ********************** *****

dgic.t1_BETA1773 ATQGTVSGLATEETSSTIVTIQEVKPPKKDEVETKDDRPSPVTNVLDSADSVYELEKVKR

dgic.t1_93161P AIQGTVSGQATEETSSTIVTIQEAKPPKKDEVETKDDRPSPVTNVLDSADSVYELEKVKR

dgic.t1_RefBeet-1.1 AIQGIVSGQAIEEASSTIVTIQEAKPPKKDEVETKDDRPSPVTNVFDSTDSVHELEKVKR

* ** *** * ** ********* ********************* ** *** *******

dgic.t1_BETA1773 EMKMMESALQGAAKQAQAKADEIAKLMNENEQLKSVVENLNRKSNGEIESLREEYHQRVA

dgic.t1_93161P EMKMMESALQGAAKQAQAKADEIAKLMNENEQLKSVVENLNRKSNGEIESLREEYHQRVA

dgic.t1_RefBeet-1.1 EMKMMETALQGAAKQAQAKADEIAKLMNENEQLKSVVENLSRKSNGEIESLREEYHQRVA

****** ********************************* *******************

dgic.t1_BETA1773 TLERKVYALTKERDTLRREQNKRSDAAALLKEKDEIINQVMAEGEELSKKQAAQEAQMRK

dgic.t1_93161P TLERKVYALTKERDTLRREQNKRSDAAALLKEKDEIINQVMAEGEELSKKQAAQEAQMRK

dgic.t1_RefBeet-1.1 TLERKVYALTKERDTLRREQNKRSDAAALLKEKDEIINQVMAEGEELSKKQAAQEAQMRK

************************************************************

dgic.t1_BETA1773 LRAQIRELDEEKKALATKLQLEENKVESIKKDKAATEKLLQETIEKHQAELAGQKEYYTN

dgic.t1_93161P LRAQIRELDEEKKALATKLQLEENKVESIKKDKAATEKLLQETIEKHQAELAGQKEYYTN

dgic.t1_RefBeet-1.1 LRAQIRELDEEKKALATKLQLEENKVESIRKDKAATEKLLQETIEKHQAELAGQKEYYTN

***************************** ******************************

dgic.t1_BETA1773 ALSAAKEAEALAEARANDEARNELEGRLREAEERDAMLVQTLEELRQTLSSQEQQAIVKE

dgic.t1_93161P ALSAAKEAEALAEARANDEARNELEGRLREAEERDAMLVQTLEELRQTLSSQEQQAIVKE

dgic.t1_RefBeet-1.1 ALNAAKEAEALAEARANDEARNELEGRLREAEERDAMLVQTLEELRQTLSSQEQQAIVKE

** *********************************************************

dgic.t1_BETA1773 DMLRRDIEDLQKRYQESERRCEELVSQLPESTRPLLRQIEAMQETSARKAEAWVAVERTL

dgic.t1_93161P DMLRRDIEDLQKRYQESERRCEELVSQLPESTRPLLRQIEAMQETSARKAEAWVAVERTL

dgic.t1_RefBeet-1.1 DMLRREIEDLQKRYQESERRCEELVSQLPESTRPLLRQIEAMQETSARKAEAWVAVERTL

***** ******************************************************

dgic.t1_BETA1773 QSRLQEAEAKAASAEERERSMNDRLSQTLSRINVLEAQISCLRAEQTQLNRSLEKERQRA

dgic.t1_93161P QSRLQEAEAKAASAEERERSMNDRLSQTLSRINVLEAQISCLRAEQTQLNRSLEKERQRA

dgic.t1_RefBeet-1.1 QSRLQEAEAKAASAEERERSMNDRLSQTLSRINVLEAQISCLRAEQTQLNRSLEKERQRA

************************************************************

dgic.t1_BETA1773 AENRQEYLAAKEIADTHEGRANQLDEEIKGLKRKHKNELQESLAHRELLQQDLDREKAAR

dgic.t1_93161P AENRQEYLAAKEIADTHEGRANQLDEEIKGLKRKHKNELQESLAHRELLQQELDREKAAR

dgic.t1_RefBeet-1.1 AENRQEYLAAKEIADTHEGRANQLDEEIKGLKRKHKNELQESLAHRELLQQELDREKAAR

*************************************************** ********

dgic.t1_BETA1773 LDLEKASHVQSSLADQSPTTKKSSTLENGMLTRKLSSASSVGNIEESFFLQASLDSSSSL

dgic.t1_93161P LDLEKAAHVQSSLADQSPTTKKSSTLENGMLTRKLSSASSVGNIEESFFLQASLDSSSSL

dgic.t1_RefBeet-1.1 LDLEKAAHVQSSLADQSPTTKKSSTHENGMLTRKLSSASSVGNIEESFFLQASLDSSSSL

****** ****************** **********************************

dgic.t1_BETA1773 LERRNSGEATMSPYYLKSMTPSAFEASLRQKEGELASYMSRLASMEAIRDSLAEELVKMT

dgic.t1_93161P LERRNSGEATMSPYYLKSMTPSAFEASLRQKEGELASYMSRLASMEAIRDSLAEELVKMT

dgic.t1_RefBeet-1.1 LERRNSGEATMSPYYLKSMTPSAFEASLRQKEGELASYMSRLASMEAIRDSLAEELVKMT

************************************************************

dgic.t1_BETA1773 EQCEKLRSEATMLPGIRAELEALRRRHTAALELMGERDEELEELRADIVDLKEMYREQVN

dgic.t1_93161P EQCEKLRSEATMLPGIRAELEALRRRHTAALELMGERDEELEELRADIVDLKEMYREQVN

dgic.t1_RefBeet-1.1 EQCEKLRSEATMLPGIRAELEALRRRHTAALELMGERDEELEELRADIVDLKEMYREQVN

************************************************************

dgic.t1_BETA1773 LLVNKIQTLSSQMGAT*

dgic.t1_93161P LLVNKIQTLSSQMGAT*

dgic.t1_RefBeet-1.1 LLVNKIQTLSSQMGAT*

*****************

## Bv9_227340_kzoy.t1

kzoy.t1_BETA1773 MANPKGSANIRNLMQNGKNSLLPPKSPFPSITPAYPDYGPNTCLGSKSALRCRDGNSYHQ

kzoy.t1_93161P MANPKGSANIRNLMQNGKNSLLPPKSPFPSITPAYPDYGPNTCLGSKSALRCRDGNSYHQ

kzoy.t1_RefBeet-1.1 MANPKGSANIRNLMQNGKNSLLPPKSPFPSITPAYPDYGPNTCLGSKSALRCRDGNSYHQ

************************************************************

kzoy.t1_BETA1773 RTSSESVIEEQPSWLDELLNEPETPVRRGHRRSSSDSFAYSDAANMSNMDYMVPLDETKF

kzoy.t1_93161P RTSSESVIEEQPSWLDELLNEPETPVRRGHRRSSSDSFAYSDAANMSNMDYMVPLDETKF

kzoy.t1_RefBeet-1.1 RTSSESVIEEQPSWLDELLNEPETPVRRGHRRSSSDSFAYSDAANMSNMDYMVPLDETKF

************************************************************

kzoy.t1_BETA1773 KSMVSSSLWGSQNFDLSKETLQGSFHHDSNFYGKLRNRSLELKLAPSNHTNGLRSSRDKA

kzoy.t1_93161P KSMVQSSLWGSQNFDLSKETLQGSFHHDSNFYGKLRNRSLELKLAPSNHTNGLRSSRDKA

kzoy.t1_RefBeet-1.1 KSMVSSSLWGSQNFDLSKETLQGSFHHDSNFYGKLRNRSLELKLAPSNHTNGLRSSRDKA

**** *******************************************************

kzoy.t1_BETA1773 VTQSAGASAAALELDSVPSTAAEKLDQSESGSHDPKSFFEKKDGSHAKTPSENDTKRAKQ

kzoy.t1_93161P VTQSAGASAAALELDSVPSTAAEKLDQSESGSHDPKSFFEKKDGSHAKTPSENDTKRAKQ

kzoy.t1_RefBeet-1.1 VTQSAGASAAALELDSIPSTAAEKLDQSESGSHDPKSFFEKKDGSHAKTPSENDTKRAKQ

**************** *******************************************

kzoy.t1_BETA1773 QFAQRSRVRKLQYIAELERSVQSLQAEGSEVSAELEFLNQQNLILSMENKALKQRLESLA

kzoy.t1_93161P QFAQRSRVRKLQYIAELERSVQSLQAEGSEVSAELEFLNQQNLILSMENKALKQRLESLA

kzoy.t1_RefBeet-1.1 QFAQRSRVRKLQYIAELERSVQSLQAEGSEVSAELEFLNQQNLILSMENKALKQRLESLA

************************************************************

kzoy.t1_BETA1773 QEKLIKYLEHEVLEREIGRLRALYQQQQQQSQLQPQPHQRPSSSHRRTNSRDLDAQFANL

kzoy.t1_93161P QEKLIKYLEHEVLEREIGRLRALYQQQQQQSQLQPQPHQRPSSSHRRTNSRDLDAQFANL

kzoy.t1_RefBeet-1.1 QEKLIKYLEHEVLEREIGRLRALYQQQQQQSQLQPQPHQRPSSSHRRTNSRDLDAQFANL

************************************************************

kzoy.t1_BETA1773 GLKHKETSSGADPFSGPVGI*

kzoy.t1_93161P GLKHKETSSGADPFSGPVGI*

kzoy.t1_RefBeet-1.1 GLKHKETSSGADPFSGPVGI*

*********************

## Bv9_227350_pgzt.t1

pgzt.t1_BETA1773 MGKNPLSFRRTISRRRNNRKVVPPENSAIEIVPTTKNDDVAGTSASKGKKKMGGARLWMK

pgzt.t1_93161P MGKNPLSFRRTISRRRNNRKVVPPENSAIEIVPTTKNDDVAGTSASKGKKKMGGARLWMK

pgzt.t1_RefBeet-1.1 MGKNPLSFRRTISRRRNNRKVVPPENSAIEIVPTTKNDDVAGTSASKGKKKMGGARLWMK

************************************************************

pgzt.t1_BETA1773 FDKFGQSELIEWDKSAIIKRVGIPARDLRILGPVFSQSSNILARERAMVVNLEFIKAIVT

pgzt.t1_93161P FDKFGQSELIEWDKSAIIKRVGIPARDLRILGPVFSQSSNILARERAMVVNLEFIKAIVT

pgzt.t1_RefBeet-1.1 FDKFGQSELIEWDKSAIIKRVGIPARDLRILGPVFSQSSNILARERAMVVNLEFIKAIVT

************************************************************

pgzt.t1_BETA1773 AEEVLLLDPLRQEVLPFVDQLRQQLPQKSQGMNKAGHVDDRDSKQHPTEVQWARTPEPVE

pgzt.t1_93161P AEEVLLLDPLRQEVLPFVDQLRQQLPQKSQGMNKAGHVDDRDSKQHPTEVQWARTPEPVE

pgzt.t1_RefBeet-1.1 AEEVLLLDPLRQEVLPFVDQLRQQLPQKSQGMNKAGHVDDGDSKQHPTEVQWARTPEPVE

**************************************** *******************

pgzt.t1_BETA1773 GEQQELPFEFQVLEIALEVVCSYLDSNVAELEKHAYPVLDDLARNVSTKNLKRVRSLKSN

pgzt.t1_93161P GEQQELPFEFQVLEIALEVVCSYLDSNVAELEKHAYPVLDDLARNVSTKNLKRVRSLKSN

pgzt.t1_RefBeet-1.1 GEQQELPFEFQVLEIALEVVCSYLDSNVAELEKHAYPVLDDLARNVSTKNLKRVRSLKSN

************************************************************

pgzt.t1_BETA1773 LTRLLARVQKVRDEIEHLLDDNEDMAHLYLTRKLIQYQQSEALLNSAASNSIVATPHALH

pgzt.t1_93161P LTRLLARVQKVRDEIEHLLDDNEDMAHLYLTRKLIQYQQSEALLNSAASNSIVATPHALH

pgzt.t1_RefBeet-1.1 LTRLLARVQKVRDEIEHLLDDNEDMAHLYLTRKLIQYQQSEALLNSAASNSIVPTPHVLH

***************************************************** ***.**

pgzt.t1_BETA1773 RNSSYRSGSMMTSNYWDDDVEDLEMLLEAYFMQLDGTRNKILSVREYIDDTEDYVNIQLD

pgzt.t1_93161P RNSSYRSGSMMTSNYWDDDVEDLEMLLEAYFMQLDGTRNKILSVREYIDDTEDYVNIQLD

pgzt.t1_RefBeet-1.1 RNSSYRSGSMMTSNYWDDDVEDLEMLLEAYFMQLDGTRNKILSVREYIDDTEDYVNIQLD

************************************************************

pgzt.t1_BETA1773 NQRNELIQLQLNLTIASFAIAIDTLIAGAFGMNIPCSLYKTNGVFGYVVGGGTAFSFLIF

pgzt.t1_93161P NQRNELIQLQLNLTIASFAIAIDTLIAGAFGMNIPCSLYKTNGVFGYVVGGGTAFSFLIF

pgzt.t1_RefBeet-1.1 NQRNELIQLQLNLTIASFAIAIDTLIAGAFGMNIPCSLYKTNGVFGYVVGGGTAFSFLIF

************************************************************

pgzt.t1_BETA1773 ILILGYARWKKLLGS*

pgzt.t1_93161P ILILGYARWKKLLGS*

pgzt.t1_RefBeet-1.1 ILILGYARWKKLLGS*

****************

## Bv9_227360_oeyr.t1

oeyr.t1_BETA1773 MASIVQPPSSSLKKRDTPASREGDQLIITPLGAGNEVGRSCVYMSYKGKTVLFDCGIHPA

oeyr.t1_93161P MASIVQPPSSSLKKRDSPASREGDQLIITPLGAGNEVGRSCVYMSYKGKTVLFDCGIHPA

oeyr.t1_RefBeet-1.1 MASIVQPPSSSLKKRDTPASREGDQLIITPLGAGNEVGRSCVYMSYKGKTVLFDCGIHPA

****************:*******************************************

oeyr.t1_BETA1773 YSGMAALPYFDEIDPSTIDVLLVTHFHLDHAASLPYFLEKTTFKGKVYMTHATKAIYRLL

oeyr.t1_93161P YSGMAALPYFDEIDPSTIDVLLVTHFHLDHAASLPYFLEKTTFKGKVYMTHATKAIYRLL

oeyr.t1_RefBeet-1.1 YSGMAALPYFDEIDPSTIDVLLVTHFHLDHAASLPYFLEKTTFKGKVYMTHATKAIYRLL

************************************************************

oeyr.t1_BETA1773 LSDYVKVSKVSVEDMLFDEQDILRSMDRIEVIDFHQTLEVNGIRFWCYTAGHVLGAAMFM

oeyr.t1_93161P LSDYVKVSKVSVEDMLFDEQDILRSMDRIEVIDFHQTLEVNGIRFWCYTAGHVLGAAMFM

oeyr.t1_RefBeet-1.1 LSDYVKVSKVSVEDMLFDEQDILRSMDRIEVIDFHQTLEVNGIRFWCYTAGHVLGAAMFM

************************************************************

oeyr.t1_BETA1773 VDIAGVRVLYTGDYSREEDRHLRSAETPQFSPDICIIESTYGVQLHQPRHVREKRFTDVI

oeyr.t1_93161P VDIAGVRVLYTGDYSREEDRHLRSAETPQFSPDICIIESTYGVQLHQPRHVREKRFTDVI

oeyr.t1_RefBeet-1.1 VDIAGVRVLYTGDYSREEDRHLRSAETPQFSPDICIIESTYGVQLHQPRHVREKRFTDVI

************************************************************

oeyr.t1_BETA1773 HSTVAQGGRVLIPAFALGRAQELLLILDEYWSNHPELHNIPIYYASPLAKRCMAVYQTYI

oeyr.t1_93161P HSTVAQGGRVLIPAFALGRAQELLLILDEYWSNHPELHNIPIYYASPLAKRCMAVYQTYI

oeyr.t1_RefBeet-1.1 HSTVAQGGRVLIPAFALGRAQELLLILDEYWSNHPELHNIPIYYASPLAKRCMAVYQTYI

************************************************************

oeyr.t1_BETA1773 NAMNDRIRNQFANSNPFDFKHISPLKSIENFDDVGPSVVMASPGGLQSGLSRQLFDKWCS

oeyr.t1_93161P NAMNDRIRNQFANSNPFDFKHISPLKSIENFDDVGPSVVMASPGGLQSGLSRQLFDKWCS

oeyr.t1_RefBeet-1.1 NAMNDRIRNQFANSNPFDFKHISPLKSIENFDDVGPSVVMASPGGLQSGLSRQLFDKWCS

************************************************************

oeyr.t1_BETA1773 DKKNSCVIPGYVVEGTLAKTIINEPKEVTLMNGLTAPLNMQVHYISFSAHADYSETSAFL

oeyr.t1_93161P DKKNSCVIPGYVVEGTLAKTIINEPKEVTLMNGLTAPLNMQVHYISFSAHADYSETSAFL

oeyr.t1_RefBeet-1.1 DKKNSCVIPGYVVEGTLAKTIINEPKEVTLMNGLTAPLNMQVHYISFSAHADYSETSAFL

************************************************************

oeyr.t1_BETA1773 KELMPPNIILVHGEANEMGRLKQKLISLFSDRNTKIMSPKNCQSVEMSFSSEKMAKTIGR

oeyr.t1_93161P KELMPPNIILVHGEANEMGRLKQKLISLFSDRNTKIMSPKNCQSVEMSFSSEKMAKTIGR

oeyr.t1_RefBeet-1.1 KELMPPNIILVHGEANEMGRLKQKLISLFSDRNTKIMSPKNCQSVEMSFSSEKMAKTIGR

************************************************************

oeyr.t1_BETA1773 LAEKTPEVGESVSGLLVKKGFTYQIMAPEDLHVFSQLCTANVNQRITIPYSGAFGVLKHR

oeyr.t1_93161P LAEKTPEVGESVSGLLVKKGFTYQIMAPEDLHVFSQLCTANVNQRITIPYSGAFGVLKHR

oeyr.t1_RefBeet-1.1 LAEKTPEVGESVSGLLVKKGFTYQIMAPEDLHVFSQLCTANVNQRITIPYSGAFGVLKHR

************************************************************

oeyr.t1_BETA1773 LRQIYESVESGVDEDSDVPTLQVHGSVTMKQESEKHISLHWTADPISDMVSDSIVAMVLN

oeyr.t1_93161P LRQIYESVESGVDEDSDVPTLQVHGSVTMKQESEKHISLHWTADPISDMVSDSIVAMVLN

oeyr.t1_RefBeet-1.1 LRQIYESVESGVDEDSDVPTLQVHGSVTMKQESEKHISLHWTADPISDMVSDSIVAMVLN

************************************************************

oeyr.t1_BETA1773 MSKEMPKVVVESDSERTKEENQKKMEKVVHALLVSLFGDVKLGEDGRLVITVDNNVAYLD

oeyr.t1_93161P MSKEMPKVVVESDSERTEEENQKKMEKVAHALLVSLFGDVKLGEDGRLVITVDNNVAYLD

oeyr.t1_RefBeet-1.1 MSKEMPKVVVESDSERTEEENQKKMEKVAHALLVSLFGDVKLGEDGRLVITVDNNVAYLD

*****************:**********.*******************************

oeyr.t1_BETA1773 KQTGDVESENEGLKERVRVAFRRIDSAVKPIPLPAA*

oeyr.t1_93161P KQTGDVESDNEGLKERVRVAFRRIDSAVKPIPLPAA*

oeyr.t1_RefBeet-1.1 KQTGDVESENEGLKERVRVAFRRIDSAVKPIPLPAA*

********:****************************

## Bv9_227370_yfgr.t1

yfgr.t1_BETA1773 MAALPYFDEIDPSTIDVVLVTHFHLDHAASLPYFLEKTTFKGKVYMTHATKAIYRLLLSD

yfgr.t1_93161P MAALPYFDEIDPSTIDVVLVTHFHLDHAASLPYFLEKTTFKGKVYMTHATKAIYRLLLSD

yfgr.t1_RefBeet-1.1 MAALPYFDEIDPSTIDVVLVTHFHLDHAASLPYFLEKTTFKGKVYMTHATKAIYRLLLSD

************************************************************

yfgr.t1_BETA1773 YVKVSKVSVEDMLFDEQDILRSMDRIEVIDFHQTLEVNGIRFWCYTAGHVLGAAMFMVDI

yfgr.t1_93161P YVKVSKVSVEDMLFDEQDILRSMDRIEVIDFHQTLEVNGIRFWCYTAGHVLGAAMFMVDI

yfgr.t1_RefBeet-1.1 YVKVSKVSVEDMLFDEQDILRSMDRIEVIDFHQTLEVNGIRFWCYTAGHVLGAAMFMVDI

************************************************************

yfgr.t1_BETA1773 AGVRVLYTGDFSREEDRHLRSAEAPQFSPDICIIESTYGVQLHQPRHVREKRFTDVIHST

yfgr.t1_93161P AGVRVLYTGDFSREEDRHLRSAETPQFSPDICIIESTYGVQLHQPRHVREKRFTDVIHST

yfgr.t1_RefBeet-1.1 AGVRVLYTGDFSREEDRHLRSAETPQFSPDICIIESTYGVQLHQPRHVREKRFTDVIHST

***********************:************************************

yfgr.t1_BETA1773 VAQGGRVLIPACPWSCPGTIINP*------------------------------------

yfgr.t1_93161P VAQGGRVLIPAYALGRAQELLLILDEYWSNHPELHNIPIYYASPLAKRCMAVYQTYINAM

yfgr.t1_RefBeet-1.1 VAQGGRVLIPAYALGRAQELLLILDEYWSNHHELHNIPIYYASPLAKRCMAVYQTYINAM

*********** . ::

yfgr.t1_BETA1773 ------------------------------------------------------------

yfgr.t1_93161P NDRIRNQFANSNPFDFKHISPLKSIENFDDVGPSVVMASPGGLQSGLSRQLFDKWCSDKK

yfgr.t1_RefBeet-1.1 NDRIRNQFANSNPFDFKHISPLKSIENFDDVGPSVVMASPGGLQSGLSRQLFDKWCSDKK

yfgr.t1_BETA1773 ------------------------------------------------------------

yfgr.t1_93161P NSCVIPGYVVDGTLAKTIINEPKEVTLMNGLTAPLNMQVHYISFSAHADYSETSAFLKEL

yfgr.t1_RefBeet-1.1 NSCVIPGYVVDGTLAKTIINEPKEVTLMNGLTAPLNMQVHYISFSAHADYSETSAFLKEL

yfgr.t1_BETA1773 ------------------------------------------------------------

yfgr.t1_93161P MPPNIILVHGGANEMGRLKQKLTSLFSDHNTKIMSPKNCQSVEMSFSSEKMAKTIGRLAE

yfgr.t1_RefBeet-1.1 MPPNIILVHGGANEMGRLKQKLTSLFSDHNTKIMSPKNCQSVEMSFSSEKMAKTIGRLAE

yfgr.t1_BETA1773 ------------------------------------------------------------

yfgr.t1_93161P KTPEIGESVSGLLVKKGFTYQIMAPEDLHIFSQLCTSNVNQRITIPYSGAFGVLKHRLKQ

yfgr.t1_RefBeet-1.1 KTPEIGESVSGLLVKKGFTYQIMAPEDLHIFSQLCTSNVNQRITIPYSGAFGVLKHRLKQ

yfgr.t1_BETA1773 ------------------------------------------------------------

yfgr.t1_93161P IYESVESGVDEDSNVPTLQVHGSVTTKQESKKHISLHWTADPISDMVSDSIVAMVLNMSK

yfgr.t1_RefBeet-1.1 IYESVESGVDEDSNVPTLQVHGSVTTKQESKKHISLHWTADPISDMVSDSIVAMVLNMSK

yfgr.t1_BETA1773 ------------------------------------------------------------

yfgr.t1_93161P EMPKVVVESDNSERSEEEKQKMEKVAHALLVSLFGDVKLGEDGRLVITIDNNVAYLDKQT

yfgr.t1_RefBeet-1.1 EMPKVVVESDNSERSEEEKQKMEKVAHALLVSLFGDVKLGEDGRLVITIDNNVAYLDKQT

yfgr.t1_BETA1773 ----------------------------------

yfgr.t1_93161P GDVESENEGLKERVRVAFRRIDSAVKPIPLPAA*

yfgr.t1_RefBeet-1.1 GDVESENEGLKERVRVAFRRIDSAVKPIPLPAA*

## Bv9_227360_oeyr.t1 and Bv9_227370_yfgr.t1

yfgr.t1_BETA1773 ------------------------------------------------------------

yfgr.t1_93161P ------------------------------------------------------------

yfgr.t1_RefBeet-1.1 ------------------------------------------------------------

oeyr.t1_BETA1773 MASIVQPPSSSLKKRDTPASREGDQLIITPLGAGNEVGRSCVYMSYKGKTVLFDCGIHPA

oeyr.t1_93161P MASIVQPPSSSLKKRDSPASREGDQLIITPLGAGNEVGRSCVYMSYKGKTVLFDCGIHPA

oeyr.t1_RefBeet-1.1 MASIVQPPSSSLKKRDTPASREGDQLIITPLGAGNEVGRSCVYMSYKGKTVLFDCGIHPA

yfgr.t1_BETA1773 ---MAALPYFDEIDPSTIDVVLVTHFHLDHAASLPYFLEKTTFKGKVYMTHATKAIYRLL

yfgr.t1_93161P ---MAALPYFDEIDPSTIDVVLVTHFHLDHAASLPYFLEKTTFKGKVYMTHATKAIYRLL

yfgr.t1_RefBeet-1.1 ---MAALPYFDEIDPSTIDVVLVTHFHLDHAASLPYFLEKTTFKGKVYMTHATKAIYRLL

oeyr.t1_BETA1773 YSGMAALPYFDEIDPSTIDVLLVTHFHLDHAASLPYFLEKTTFKGKVYMTHATKAIYRLL

oeyr.t1_93161P YSGMAALPYFDEIDPSTIDVLLVTHFHLDHAASLPYFLEKTTFKGKVYMTHATKAIYRLL

oeyr.t1_RefBeet-1.1 YSGMAALPYFDEIDPSTIDVLLVTHFHLDHAASLPYFLEKTTFKGKVYMTHATKAIYRLL

*****************:***************************************

yfgr.t1_BETA1773 LSDYVKVSKVSVEDMLFDEQDILRSMDRIEVIDFHQTLEVNGIRFWCYTAGHVLGAAMFM

yfgr.t1_93161P LSDYVKVSKVSVEDMLFDEQDILRSMDRIEVIDFHQTLEVNGIRFWCYTAGHVLGAAMFM

yfgr.t1_RefBeet-1.1 LSDYVKVSKVSVEDMLFDEQDILRSMDRIEVIDFHQTLEVNGIRFWCYTAGHVLGAAMFM

oeyr.t1_BETA1773 LSDYVKVSKVSVEDMLFDEQDILRSMDRIEVIDFHQTLEVNGIRFWCYTAGHVLGAAMFM

oeyr.t1_93161P LSDYVKVSKVSVEDMLFDEQDILRSMDRIEVIDFHQTLEVNGIRFWCYTAGHVLGAAMFM

oeyr.t1_RefBeet-1.1 LSDYVKVSKVSVEDMLFDEQDILRSMDRIEVIDFHQTLEVNGIRFWCYTAGHVLGAAMFM

************************************************************

yfgr.t1_BETA1773 VDIAGVRVLYTGDFSREEDRHLRSAEAPQFSPDICIIESTYGVQLHQPRHVREKRFTDVI

yfgr.t1_93161P VDIAGVRVLYTGDFSREEDRHLRSAETPQFSPDICIIESTYGVQLHQPRHVREKRFTDVI

yfgr.t1_RefBeet-1.1 VDIAGVRVLYTGDFSREEDRHLRSAETPQFSPDICIIESTYGVQLHQPRHVREKRFTDVI

oeyr.t1_BETA1773 VDIAGVRVLYTGDYSREEDRHLRSAETPQFSPDICIIESTYGVQLHQPRHVREKRFTDVI

oeyr.t1_93161P VDIAGVRVLYTGDYSREEDRHLRSAETPQFSPDICIIESTYGVQLHQPRHVREKRFTDVI

oeyr.t1_RefBeet-1.1 VDIAGVRVLYTGDYSREEDRHLRSAETPQFSPDICIIESTYGVQLHQPRHVREKRFTDVI

*************:************:*********************************

yfgr.t1_BETA1773 HSTVAQGGRVLIPACPWSCPGTIINP*---------------------------------

yfgr.t1_93161P HSTVAQGGRVLIPAYALGRAQELLLILDEYWSNHPELHNIPIYYASPLAKRCMAVYQTYI

yfgr.t1_RefBeet-1.1 HSTVAQGGRVLIPAYALGRAQELLLILDEYWSNHHELHNIPIYYASPLAKRCMAVYQTYI

oeyr.t1_BETA1773 HSTVAQGGRVLIPAFALGRAQELLLILDEYWSNHPELHNIPIYYASPLAKRCMAVYQTYI

oeyr.t1_93161P HSTVAQGGRVLIPAFALGRAQELLLILDEYWSNHPELHNIPIYYASPLAKRCMAVYQTYI

oeyr.t1_RefBeet-1.1 HSTVAQGGRVLIPAFALGRAQELLLILDEYWSNHPELHNIPIYYASPLAKRCMAVYQTYI

************** . ::

yfgr.t1_BETA1773 ------------------------------------------------------------

yfgr.t1_93161P NAMNDRIRNQFANSNPFDFKHISPLKSIENFDDVGPSVVMASPGGLQSGLSRQLFDKWCS

yfgr.t1_RefBeet-1.1 NAMNDRIRNQFANSNPFDFKHISPLKSIENFDDVGPSVVMASPGGLQSGLSRQLFDKWCS

oeyr.t1_BETA1773 NAMNDRIRNQFANSNPFDFKHISPLKSIENFDDVGPSVVMASPGGLQSGLSRQLFDKWCS

oeyr.t1_93161P NAMNDRIRNQFANSNPFDFKHISPLKSIENFDDVGPSVVMASPGGLQSGLSRQLFDKWCS

oeyr.t1_RefBeet-1.1 NAMNDRIRNQFANSNPFDFKHISPLKSIENFDDVGPSVVMASPGGLQSGLSRQLFDKWCS

yfgr.t1_BETA1773 ------------------------------------------------------------

yfgr.t1_93161P DKKNSCVIPGYVVDGTLAKTIINEPKEVTLMNGLTAPLNMQVHYISFSAHADYSETSAFL

yfgr.t1_RefBeet-1.1 DKKNSCVIPGYVVDGTLAKTIINEPKEVTLMNGLTAPLNMQVHYISFSAHADYSETSAFL

oeyr.t1_BETA1773 DKKNSCVIPGYVVEGTLAKTIINEPKEVTLMNGLTAPLNMQVHYISFSAHADYSETSAFL

oeyr.t1_93161P DKKNSCVIPGYVVEGTLAKTIINEPKEVTLMNGLTAPLNMQVHYISFSAHADYSETSAFL

oeyr.t1_RefBeet-1.1 DKKNSCVIPGYVVEGTLAKTIINEPKEVTLMNGLTAPLNMQVHYISFSAHADYSETSAFL

yfgr.t1_BETA1773 ------------------------------------------------------------

yfgr.t1_93161P KELMPPNIILVHGGANEMGRLKQKLTSLFSDHNTKIMSPKNCQSVEMSFSSEKMAKTIGR

yfgr.t1_RefBeet-1.1 KELMPPNIILVHGGANEMGRLKQKLTSLFSDHNTKIMSPKNCQSVEMSFSSEKMAKTIGR

oeyr.t1_BETA1773 KELMPPNIILVHGEANEMGRLKQKLISLFSDRNTKIMSPKNCQSVEMSFSSEKMAKTIGR

oeyr.t1_93161P KELMPPNIILVHGEANEMGRLKQKLISLFSDRNTKIMSPKNCQSVEMSFSSEKMAKTIGR

oeyr.t1_RefBeet-1.1 KELMPPNIILVHGEANEMGRLKQKLISLFSDRNTKIMSPKNCQSVEMSFSSEKMAKTIGR

yfgr.t1_BETA1773 ------------------------------------------------------------

yfgr.t1_93161P LAEKTPEIGESVSGLLVKKGFTYQIMAPEDLHIFSQLCTSNVNQRITIPYSGAFGVLKHR

yfgr.t1_RefBeet-1.1 LAEKTPEIGESVSGLLVKKGFTYQIMAPEDLHIFSQLCTSNVNQRITIPYSGAFGVLKHR

oeyr.t1_BETA1773 LAEKTPEVGESVSGLLVKKGFTYQIMAPEDLHVFSQLCTANVNQRITIPYSGAFGVLKHR

oeyr.t1_93161P LAEKTPEVGESVSGLLVKKGFTYQIMAPEDLHVFSQLCTANVNQRITIPYSGAFGVLKHR

oeyr.t1_RefBeet-1.1 LAEKTPEVGESVSGLLVKKGFTYQIMAPEDLHVFSQLCTANVNQRITIPYSGAFGVLKHR

yfgr.t1_BETA1773 ------------------------------------------------------------

yfgr.t1_93161P LKQIYESVESGVDEDSNVPTLQVHGSVTTKQESKKHISLHWTADPISDMVSDSIVAMVLN

yfgr.t1_RefBeet-1.1 LKQIYESVESGVDEDSNVPTLQVHGSVTTKQESKKHISLHWTADPISDMVSDSIVAMVLN

oeyr.t1_BETA1773 LRQIYESVESGVDEDSDVPTLQVHGSVTMKQESEKHISLHWTADPISDMVSDSIVAMVLN

oeyr.t1_93161P LRQIYESVESGVDEDSDVPTLQVHGSVTMKQESEKHISLHWTADPISDMVSDSIVAMVLN

oeyr.t1_RefBeet-1.1 LRQIYESVESGVDEDSDVPTLQVHGSVTMKQESEKHISLHWTADPISDMVSDSIVAMVLN

yfgr.t1_BETA1773 ------------------------------------------------------------

yfgr.t1_93161P MSKEMPKVVVESDNSERSEEEKQKMEKVAHALLVSLFGDVKLGEDGRLVITIDNNVAYLD

yfgr.t1_RefBeet-1.1 MSKEMPKVVVESDNSERSEEEKQKMEKVAHALLVSLFGDVKLGEDGRLVITIDNNVAYLD

oeyr.t1_BETA1773 MSKEMPKVVVESDSERTKEENQKKMEKVVHALLVSLFGDVKLGEDGRLVITVDNNVAYLD

oeyr.t1_93161P MSKEMPKVVVESDSERTEEENQKKMEKVAHALLVSLFGDVKLGEDGRLVITVDNNVAYLD

oeyr.t1_RefBeet-1.1 MSKEMPKVVVESDSERTEEENQKKMEKVAHALLVSLFGDVKLGEDGRLVITVDNNVAYLD

yfgr.t1_BETA1773 -------------------------------------

yfgr.t1_93161P KQTGDVESENEGLKERVRVAFRRIDSAVKPIPLPAA*

yfgr.t1_RefBeet-1.1 KQTGDVESENEGLKERVRVAFRRIDSAVKPIPLPAA*

oeyr.t1_BETA1773 KQTGDVESENEGLKERVRVAFRRIDSAVKPIPLPAA*

oeyr.t1_93161P KQTGDVESDNEGLKERVRVAFRRIDSAVKPIPLPAA*

oeyr.t1_RefBeet-1.1 KQTGDVESENEGLKERVRVAFRRIDSAVKPIPLPAA*

## Bv9_227380_faqz.t1

faqz.t1_BETA1773 MRGRQAVGDAAVSTYKRVASKDRLDRLDGRIQGLTKEGGNPPWRKSLPHILVATIASFLF

faqz.t1_93161P MRGRQAVGDAAVSTYKRVASKDRLDRLDGRLQGLAKEGGNPPWRKSLPHILVATIASFLF

faqz.t1_RefBeet-1.1 MRGRQAVGDAAVSTYKRVASKDRLDRLDGRLQGLTKEGGNPPWRKSLPHILVATIASFLF

******************************:***:*************************

faqz.t1_BETA1773 GYHLGVVNDTLESISFDLGFSGNTMAEGLVVSMSLGGAFVGSLFSGSIADYFGRRRSFQL

faqz.t1_93161P GYHLGVVNDTLESISFDLGFSGNTMAEGLVVSMSLGGAFVGSLFSGSIADYFGRRRSFQL

faqz.t1_RefBeet-1.1 GYHLGVVNDTLESISFDLGFSGNTMAEGLVVSMSLGGAFVGSLFSGSIADYFGRRRSFQL

************************************************************

faqz.t1_BETA1773 CSLPMIIGASMSATTSSLMGMLTGRFLVGIGMGLGPAVAALYVAEVAPTYVRGTFGSFTQ

faqz.t1_93161P CSLPMIIGASMSATTSSLMGMLTGRFLVGIGMGLGPAVAALYVAEVAPTYVRGTFGSFTQ

faqz.t1_RefBeet-1.1 CSLPMIIGASMSATTGSLMGMLTGRFLVGIGMGLGPAVAALYVAEVAPTYVRGTFGSFTQ

***************.********************************************

faqz.t1_BETA1773 IATCLGLMASFLIGVPAKETKDWWRVCFWVPVVPAALLAISMELCAESPHWLFKKGRSVE

faqz.t1_93161P IATCLGLMASFLIGVPAKETKDWWRVCFWVPVVPAALLAISMELCAESPHWLFKKGRSVE

faqz.t1_RefBeet-1.1 IATCLGLMASFLIGVPAKETKDWWRVCFWVPVVPAALLAISMELCAESPHWLFKKGRSVE

************************************************************

faqz.t1_BETA1773 AEAEFERVLGGLHVKSAIAELSKSDRVDEADTVKFLELFYGRHYKVVFIGSVLFALQQLS

faqz.t1_93161P AEAEFERVLGGLHVKSAIAELSKSDRVDEADTVKFLELFYGRHYKVVFIGSVLFALQQLS

faqz.t1_RefBeet-1.1 AEAEFERVLGGLHVKSAIAELSKSDRVDEADTVKFLELFYGRHYKVVFIGSVLFALQQLS

************************************************************

faqz.t1_BETA1773 GINAVFYFSSSVFKSAGVPPDTANMFVAIINLSGSCTATILMDKLGRKVLLLGSFLGMAV

faqz.t1_93161P GINAVFYFSSSVFKSAGVPPDTANMFVAIINLSGSCTATILMDKLGRKVLLLGSFLGMAV

faqz.t1_RefBeet-1.1 GINAVFYFSSSVFKSAGVPPDTANMFVAIINLSGSCTATILMDKLGRKVLLLGSFLGMAV

************************************************************

faqz.t1_BETA1773 ALVLQIIAACSFVSPATSVYLSVGGVLLVVLTFSLGAGPVPSLLLSEMFPNRIRAKAMAW

faqz.t1_93161P ALVLQIIAACSFVSPATSVYLSVGGVLLVVLTFSLGAGPVPSLLLSEMFPNRIRAKAMAW

faqz.t1_RefBeet-1.1 ALVLQIIAACSFVSPATSVYLSVGGVLLVVLTFSLGAGPVPSLLLSEMFPNRIRAKAMAW

************************************************************

faqz.t1_BETA1773 CMAVHWGVNFFVGLLFLRLLEQLGPKLLYTFFATCCLIGFAFVKKNVLETKGKSLQEIEL

faqz.t1_93161P CMAVHWGVNFFVGLLFLRLLEQLGPKLLYTFFATCCLIGFAFVKKNVLETKGKSLQEIEL

faqz.t1_RefBeet-1.1 CMAVHWGVNFFVGLLFLRLLEQLGPKLLYTFFATCCLIGFAFVKKNVLETKGKSLQEIEL

************************************************************

faqz.t1_BETA1773 ALLPA*

faqz.t1_93161P ALLPA*

faqz.t1_RefBeet-1.1 ALLPA*

******

**Figure S2.** Multiple sequence alignments of allele-specific protein sequences of genes located within the 103 kb interval of the *BR1* locus. * position with a single, fully conserved residue, : conservation between groups of strongly similar properties with scoring > 0.5 in the Gonnet PAM 250 matrix, . conservation between groups of weakly similar properties with scoring =< 0.5 in the Gonnet PAM 250 matrix.


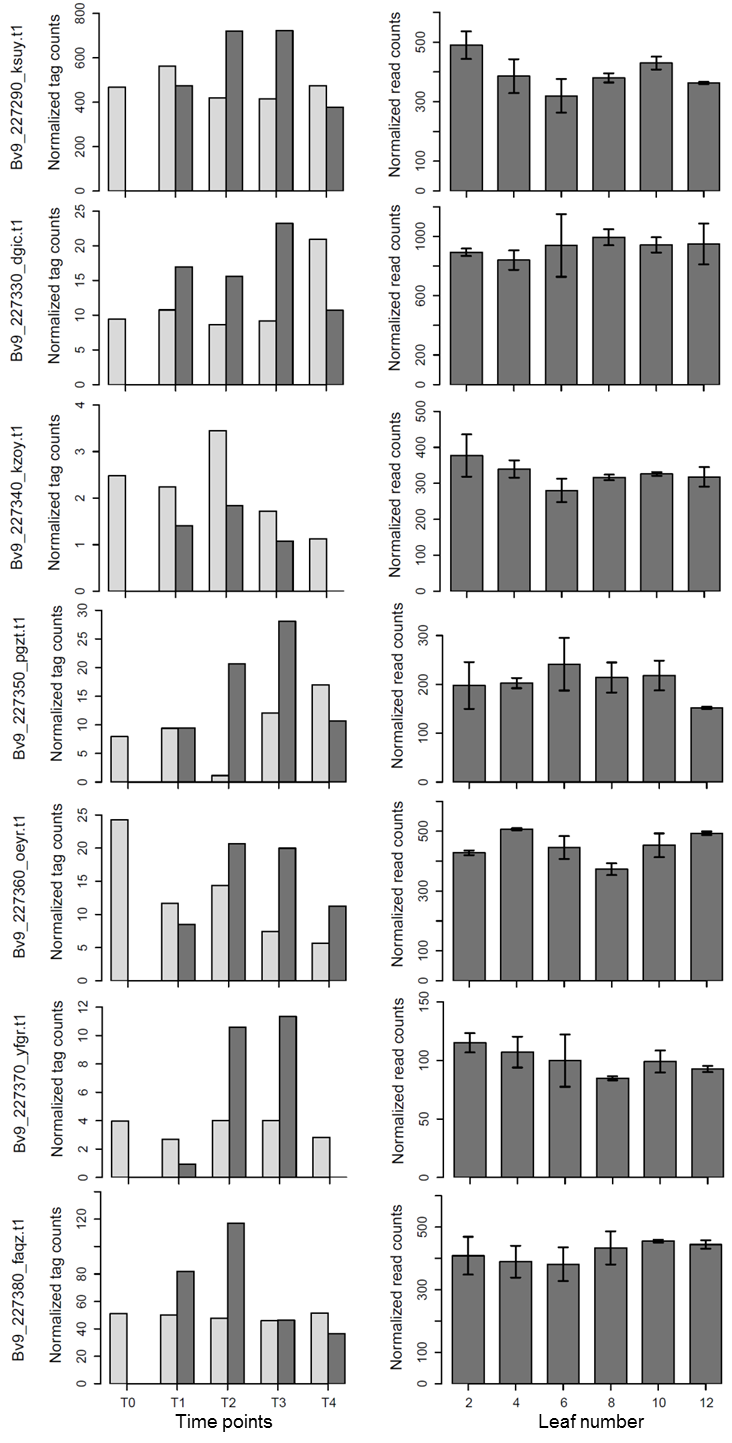


Figure S3. Transcript profiles of genes located in the *BR1* locus based on normalized tag counts of SuperSAGE data derived from leaves of non-vernalized (grey) and vernalized (dark grey) biennial sugar beets of KWS2320 at different developmental stages (left) and normalized read counts of RNAseq data derived from shoot apices of the annual sugar beet accession 001684 (right). For KWS2320, young leaves of 70 plants kept at 18°C/8 h light were harvested as mixed sample directly before cold treatment (t0), after one day (t1), 3 weeks (t2) and 12 weeks (t3) under cold treatment at 4°C/8 h light and 5 weeks after cold treatment when plants were grown at 18°C/8 h light (t4). Samples were assayed for transcript accumulation by SuperSAGE at GenXPro GmbH (Frankfurt/Main, Germany). For 001684, shoot apices of 2x 5 plants per sample grown under 16 h light were harvested at different leaf stages. Floral transition occurred during the 6 to 8-leaf stage.
